# Beta bursts spatiotemporal profiles and their links to hemodynamic responses during movement and rest

**DOI:** 10.1162/IMAG.a.1255

**Published:** 2026-05-27

**Authors:** Siyu Long, Marie-Hélène Boudrias, Georgios D. Mitsis

**Affiliations:** Integrated Program in Neuroscience, McGill University, Montréal, Canada; Center for Interdisciplinary Research in Rehabilitation of Greater Montreal (CRIR), Montréal, QC, Canada; Brain Lab, Jewish Rehabilitation Hospital, CISSS-Laval, Laval, Canada; School of Physical and Occupational Therapy, McGill University, Montréal, Canada; Department of Bioengineering, McGill University, Montréal, Canada

**Keywords:** EEG-fMRI, motor control, aperiodic components, beta bursts, hemodynamic response function, hand grips

## Abstract

Both periodic and aperiodic components in the electroencephalography (EEG) signal are known to play a role in motor control. In particular, periodic beta oscillations and their associated transient bursts (beta bursts) have been linked to motor inhibition. While the occurrence of these bursts is well-documented during simple motor tasks, their spatiotemporal distribution during more complex movements remains largely unexplored. This gap in our understanding extends to the relationship between transient EEG events and Blood Oxygenation Level Dependent (BOLD) activity, typically measured with functional magnetic resonance imaging (fMRI). To better understand these and their hemodynamic and functional correlates, simultaneous EEG and fMRI recordings were obtained at rest and during hand movements in 11 healthy adults. The spatiotemporal distribution for both aperiodic components and beta bursts was mapped during different phases of a handgrip task (low-level, ramp, and high-level grip force conditions). Additionally, the modulation of hemodynamic responses by beta bursts was investigated during both conditions. To this end, the detected beta bursts were used to estimate a hemodynamic response function (HRF) and predict the corresponding BOLD fMRI activity. During movement transition phases, a significant increase in the exponent and offset of the aperiodic components, as well as an increase in beta burst amplitude and rate were observed, as compared to sustained contractions. Furthermore, beta bursts in the contralateral/dominant motor regions of the moving hand elicited positive hemodynamic responses during movement but negative responses during rest, although the HRF features did not differ significantly between the two conditions. Other brain regions showed consistent negative hemodynamic responses across both motor tasks and resting state. These findings reveal a directional dissociation in hemodynamic responses to beta bursts between movement and rest states in motor regions, though future studies with larger sample sizes are needed to further characterize the state-dependence of this relationship. This work advances our understanding of the relationship between transient neural events and hemodynamic responses during movement-related processes in healthy individuals.

## 1 Introduction

Neural activity in the sensorimotor system is dominated by synchronized oscillations between populations of neurons, especially in the beta (13–30 Hz) frequency range (Brown, 2007). These oscillations can be recorded in the primary motor cortex (M1), primary somatosensory cortex (S1), as well as frontal and temporal cortices, where they emerge in a task-dependent manner during activities such as reaching, muscular contraction, language processing and attention tasks (Engel & Fries, 2010; Herfurth et al., 2022; Jenkinson & Brown, 2011; van Ede et al., 2011). However, cross-species studies have indicated that beta oscillations do not emerge rhythmically and continuously, but rather appear at the single-trial level in transient and discrete patterns with high amplitude, termed beta bursts (Feingold et al., 2015; Lundqvist et al., 2024; Shin et al., 2017; Tinkhauser et al., 2017). Beta bursts have been shown to be predictive markers of motor behavior, particularly in regulating movement. For instance, their crucial role in motor cancellation has been demonstrated during voluntary movements (Little & Brown, 2014; Torrecillos et al., 2018), and in action preparation following visual cues, with their occurrence correlating with movement initiation delays (Little et al., 2019). A temporal coupling between cortical beta bursts and motor unit activity has also been recently established (Bräcklein et al., 2022). Importantly, analyzing the temporal patterns of beta burst characteristics, including their amplitude, frequency, and duration, provides a framework to better understand neural variability and the state-dependent modulation of sensorimotor function (Lundqvist et al., 2024).

In addition to the aforementioned narrowband oscillatory activity, the human electroencephalography (EEG) spectrum comprises non-oscillatory (aperiodic) activity, which typically exhibits increased power within the lower frequency range (Pani et al., 2022). These aperiodic components exhibit a characteristic scale-invariant 1/f-like distribution, defined by two key parameters: the aperiodic offset, which reflects baseline power levels, and the aperiodic exponent, which characterizes the frequency-dependent power decay (Merkin et al., 2023). Neuronal activation has been shown to result in exponent flattening, where lower aperiodic exponent values indicate increased asynchronous neuronal activity (Peterson et al., 2023). The aperiodic activity is apparent in EEG spectra and can be modulated during movement execution (Pertermann et al., 2019). Notably, the electrode-averaged aperiodic exponent and offset were shown to differ significantly between resting state and motor tasks, potentially indicating a regulatory role of aperiodic signals in motor inhibitory function (Monchy et al., 2024). Recent methodological advances have contributed to removing the 1/f aperiodic characteristic from EEG time-frequency matrices, enabling more precise detection of heterogeneous beta burst subtypes (Brady & Bardouille, 2022; Szul et al., 2023). The importance of distinguishing periodic from aperiodic components in motor-related neural signals is thus becoming increasingly recognized, as it provides a more comprehensive framework for understanding the neurophysiological mechanisms underlying movement control.

Beyond beta burst modulation, movement production induces significant changes in Blood Oxygenation Level Dependent (BOLD) activity, as measured by functional magnetic resonance imaging (fMRI). Neural activity during movement triggers dynamic alterations in the ratio of deoxyhemoglobin/oxyhemoglobin concentrations due to increases in local blood flow (Frostig et al., 1990; Jasdzewski et al., 2003; Ogawa et al., 1990). For instance, when moving an object and progressively increasing muscle contraction, BOLD activation decreases in M1 and the supplementary motor area (SMA), potentially serving as a mechanism to inhibit superfluous motor output (Gazzola & Keysers, 2008; Post et al., 2009).

Simultaneous EEG-fMRI recordings have revealed temporal synchronization between electrophysiological and hemodynamic responses during rest and task conditions (Briley et al., 2021; Britz et al., 2010; Hunyadi et al., 2019; Mann-Krzisnik & Mitsis, 2022; Prokopiou et al., 2022; Walz et al., 2014). The spatial patterns of fMRI correlates associated with transient EEG network dynamics demonstrated high reproducibility and substantial overlap with conventional resting-state fMRI networks (Hunyadi et al., 2019). During task and rest periods, beta bursts have been reported to rhythmically activate task-relevant regions while simultaneously suppressing currently tonically active ones, establishing a dynamic regulatory pattern crucial for maintaining accurate sensorimotor information processing (Briley et al., 2021). In the context of movement execution, BOLD signal modulation has also been reported to show specific associations with oscillatory electrophysiological activity (Murta et al., 2015). A particularly notable example is the relationship between post-movement beta rebound (PMBR) and BOLD responses: the post-central sulcus exhibits increased BOLD signal during PMBR, with the magnitude of this hemodynamic response directly correlated with PMBR strength in sensorimotor regions (Parkes et al., 2006).

While task-dependent modulation of both periodic and aperiodic components during various motor states has been documented (Monchy et al., 2024; Torrecillos et al., 2018), previous studies have overlooked the critical distinction between pure beta bursts arising from oscillatory activity and mixed signals containing both periodic and aperiodic components. Furthermore, the temporal dynamics of beta burst patterns across different task phases and resting states remain incompletely characterized. On the other hand, simultaneous EEG-fMRI investigations have revealed concordant temporal patterns between electrophysiological and hemodynamic signals during rest and motor tasks (Britz et al., 2010; Hunyadi et al., 2019). Nevertheless, the precise relationship between transient EEG events and the corresponding BOLD fMRI hemodynamic responses remains poorly understood, particularly whether these transient events elicit positive or negative BOLD responses, as well as the precise dynamic characteristics and potential regional variability of these responses across different brain regions. Despite the established prominence of beta bursts during motor execution, their influence on motor-related BOLD activity requires further investigation. Elucidating this relationship yields promise for advancing our knowledge of neural signal integration, as well as informing the development of targeted interventions for motor learning and rehabilitation (Sporn et al., 2020; Ulanov & Shtyrov, 2022).

In this study, the spatiotemporal dynamics of periodic beta bursts and their relationship with hemodynamic responses were explored using simultaneous EEG-fMRI recordings during the resting state and hand movement. Our investigation encompassed two primary objectives. The first one was to characterize the modulation of aperiodic components and periodic beta bursts across different phases of the motor task and at rest. The second one was to quantify the beta burst-related hemodynamic response function (HRF) across brain regions. It was hypothesized that both aperiodic and periodic beta burst components would exhibit distinct patterns across different phases of motor task execution and that beta burst-related HRF effects would also exhibit distinct spatiotemporal patterns across brain regions between motor task and resting state conditions.

## 2 Methods

### 2.1 Participants

Twelve healthy volunteers (age 24.2 ± 2.8, 8 males) participated in this study after providing written informed consent, following the McGill University Ethical Advisory Committee guidelines. All participants were confirmed right-handed based on the Edinburgh Handedness Inventory (EHI) (Oldfield, 1971), with a mean EHI score of 76.7 ± 14.7 (SD) and a score range from 44.4 to 100. Data was collected at the McConnell Brain Imaging Center (BIC) of the Montreal Neurological Institute (MNI), affiliated with McGill University. Earlier findings from this dataset have been documented in other publications (Prokopiou et al., 2022; Xifra-Porxas et al., 2019).

### 2.2 Experimental paradigm

**Fig. 1.**
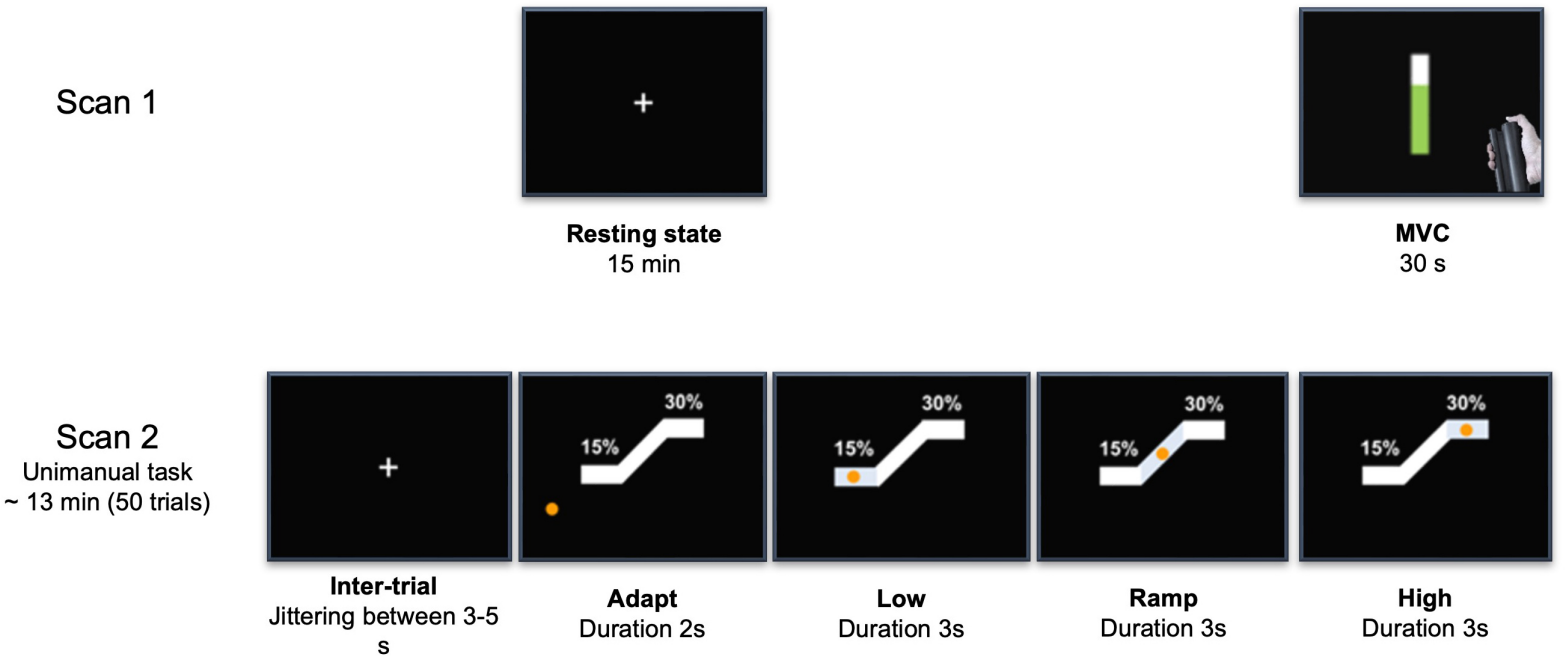
Experimental protocol: subjects underwent a resting-state experiment with eyes open (scan 1) that was subsequently followed by a unimanual (right hand) motor task (scan 2). Between the two scans, the MVC was acquired from each participant. For the unimanual motor task, in each trial subjects were initially fixated on a white crosshair, for a jittered period lasting between 3–5 seconds (inter-trial, IT). Subsequently, an orange circle appeared on the screen and subjects had to adapt their force at 15% of their MVC to reach a white vertical block and sustain their force at that level (low force level) for 3 seconds. After that, subjects had to adjust their grip force guided by a ramp to reach 30% of their MVC within a period of 3 seconds. Lastly, they had to maintain the force at that level (high force level) for another 3 seconds. A single trial lasted 11 seconds and was repeated 50 times.

Before the scans, participants completed two behavioral tests: the Nine-Hole Peg Test (NHPT) and the Box and Block Test (BBT) (Goodkin et al., 1988). Participants underwent a 15-minute simultaneously recorded EEG-fMRI scan while at eyes-open rest followed by a handgrip task performed with the dominant (right) hand. The maximum voluntary contraction (MVC) of each participant was obtained between the two scans, using the same hand gripper that was employed during the motor task. Detailed procedures are described in previous reports from our lab (Prokopiou et al., 2022; Xifra-Porxas et al., 2019). Briefly, participants performed unimanual isometric right-hand grips to track visual targets with force feedback. The motor task consisted of 50 trials of isometric grips, each comprising four phases: adapt (2 seconds, reaching 15% MVC), low force (3 seconds, maintaining 15% MVC), ramp force (3 seconds, progressively increasing to 30% MVC), and high force (3 seconds, maintaining 30% MVC), as illustrated in Figure 1. Between trials, participants rested for 3–5 seconds while fixating on a cross shown on a screen in front of them (inter-trial, IT).

### 2.3 Data acquisition

During the fMRI scanning, EEG data were continuously collected at a 5 kHz sampling rate using a 64-channel MR-compatible EEG system (Brain Products GmbH, Germany). The electrodes were placed according to the 10/20 system and referenced to electrode FCz, and they were precisely localized using a 3-D electromagnetic digitizer (Polhemus Isotrack, USA). EEG recordings were synchronized with the MRI acquisition clock using a dedicated synchronization unit to enhance MRI-related artifact correction performance. Whole-brain BOLD-fMRI signals were acquired using a 3 T MRI scanner (Siemens MAGNETOM Prisma fit) with a standard T2*-weighted EPI sequence and a 32-channel head coil. EPI sequence parameters: TR/TE = 2120/30 ms (Repetition/Echo Time), Voxel size = 3 × 3 × 4 mm, 35 slices, Slice thickness = 4 mm, Field of view (FOV) = 192 mm, Flip angle = 90°, Acquisition matrix = 64 × 64 (RO × PE), Bandwidth = 2368 Hz/Px. To facilitate the registration of functional images to a standard stereotactic space, a high-resolution T1-weighted MPRAGE structural scan was also acquired. The resting-state and motor task experiments included 420 and 400 volumes, respectively. All other acquisition parameters were identical to those previously described elsewhere (Prokopiou et al., 2022; Xifra-Porxas et al., 2019).

### 2.4 Preprocessing

The EEG data were processed offline to correct for gradient and ballistocardiogram (BCG) artifacts using BrainVision Analyser 2 (Brain Products GmbH, Germany). The gradient artifact was removed via adaptive template subtraction (Allen et al., 2000), which effectively eliminated gradient-related harmonics at 16.5, 33, and 49.5 Hz arising from the 35-slice acquisition with TR = 2120 ms, and subsequently the data were down-sampled to 1000 Hz. The data were then notch filtered at 60, 120, and 180 Hz to remove power-line artifacts. Temporal independent component analysis (ICA) was performed on each subject separately to identify and remove BCG-related artifacts (Delorme & Makeig, 2004). The BCG-related component that accounted for most of the variance in the data was isolated and used to detect heartbeat events. BCG artifacts were then removed via pulse artifact template subtraction using a moving average of EEG signals synchronized to the detected heartbeat events (Allen et al., 1998). Subsequently, the data were bandpass filtered (1–50 Hz). A second ICA decomposition was performed to reject remaining artifacts from gradient and BCG residuals, eye movements, and muscle activity. The number of preserved components averaged 21 (IQR: 13.5–23) for resting-state sessions and 16 (IQR: 13–20) for motor tasks. Following bad channel rejection, the data were re-referenced to the average reference and down-sampled to 250 Hz. One male subject was excluded from the analysis due to excessive noise. No trials were rejected to ensure signal continuity for subsequent analyses.

The fMRI data were preprocessed using FSL (version 5.0.10) (Jenkinson et al., 2012), including realignment, spatial smoothing (5 mm FWHM), high-pass temporal filtering (cutoff point = 90 seconds), and normalization to the MNI-152 template space, with a resolution of 2 mm^3^. Artifact removal was performed using spatial ICA via FSL’s MELODIC tool (Beckmann & Smith, 2004), whereby components reflecting head motion, cardiac pulsation, susceptibility artifacts, and other non-neural sources were identified and excluded based on their spatial patterns and temporal characteristics. Structural MRI processing and cortical surface reconstruction were performed using FreeSurfer (version 7.4.1), and brain parcellation was conducted using the Desikan-Killiany-Tourville (DKT) Atlas (Fischl, 2012; Klein & Tourville, 2012).

### 2.5 Source localization

The spatial resolution of 64-channel EEG has been reported to provide sufficient specificity for independent source estimation of adjacent cortical regions, including motor areas (Berki et al., 2025; Thornton et al., 2018). EEG sources were reconstructed on the cortical surface using the Brainstorm software (Tadel et al., 2011). EEG source space was reconstructed for each subject using an extension of the linearly constrained minimum variance (LCMV) beamformer (Veen et al., 1997). Data and noise covariance matrices were computed using all task phases and the resting-state broadband EEG data (1–50 Hz). For task data, the noise covariance matrix was derived from resting-state recordings of the same subject. For resting-state data, the noise covariance matrix was estimated from the diagonal elements (sensor variance values) only, following Brainstorm recommendations for resting-state EEG source reconstruction (Tadel et al., 2011). Brainstorm automatically handles rank deficiency introduced by preprocessing steps (including ICA artifact rejection) by applying regularization parameters that add a fraction of the mean variance to the diagonal of the covariance matrix to ensure numerical stability (Tadel et al., 2011). A realistic head model was obtained using each subject’s individual cortical anatomy obtained from the preprocessed structural MRI and precise electrode locations on the scalp. Lead fields were estimated using the symmetric boundary element method (BEM). The relative conductivities used for estimating the lead fields were as follows: 1 for the scalp, 0.0125 for the skull, and 1 for the brain. In total, 15,000 current dipoles were estimated on the cortical surface. The DKT Atlas was applied to parcellate all source-reconstructed data, and sources from the same parcel were combined using Principal Component Analysis (PCA). All subsequent EEG analyses were performed at the parcel level.

### 2.6 Aperiodicity analysis

Methods to extract aperiodic components, such as the fitting oscillations and one over f algorithm (FOOOF), are limited to analyzing components within a given time window, not at a single time point (Donoghue et al., 2020). A more recent algorithm (Spectral Parameterization Resolved in Time - SPRiNT) allows the decomposition of complex neural dynamics into periodic and aperiodic spectral components with high temporal resolution in the time-frequency domain (Wilson et al., 2022). The SPRiNT algorithm computes short-time Fourier transforms (STFT) for sliding time windows, converts the resulting complex frequency domain signals to power by taking the squared magnitude, and performs FOOOF fitting on the log-transformed power spectra. Here, this approach was used to estimate the aperiodic components of the EEG signal. Specifically, for the motor task data, the time series of each parcel were separated into different segments according to different task phases: inter-trial, adapt, low, ramp, and high phases. The window length for each phase matched the experimental paradigm. The SPRiNT algorithm was applied to each segment to estimate the aperiodic fit based on sliding 1-second time windows with no overlap. Subsequently, all the aperiodic fitted data were averaged across time windows, and the beta frequency band (13–30 Hz) aperiodic power spectrum was averaged across the entire frequency range for each phase to calculate the aperiodic power baseline. The same steps were performed for the resting state data analysis. The entire time series was split into 3-second phases, and the SPRiNT algorithm was performed on each phase. Additionally, the aperiodic exponent and the offset from each single phase were extracted. Finally, each phase’s time-frequency matrix was calculated using Morlet wavelets with 1 Hz central frequency and a 3 second time resolution. To assess the reliability of the SPRiNT approach, we evaluated the model fit quality across all experimental conditions. The quality of aperiodic component fitting was consistently high across all conditions (*R²* > 0.71, representing the correlation between original power spectra and fitted model spectra) with no significant differences between phases, ensuring reliable and accurate parameter estimation for all subsequent analyses (Supplementary Fig. S1).

### 2.7 Beta burst detection and characteristics calculation

The beta frequency band (13–30 Hz) was averaged across frequencies to obtain a single time series for each region and each subject representing beta oscillations. The aperiodic baseline estimated for each phase was subtracted from the corresponding time-frequency matrices to extract beta bursts. Values less than zero were set to zero after subtraction. For each phase, the threshold for beta burst extraction was set to twice the standard deviation of each single trial’s time-frequency amplitude after subtraction (Szul et al., 2023). All the phase-by-phase time-frequency matrices were binarized and concatenated following the temporal sequence of task phases (inter-trial, adaptation, low, ramp and high). Only continuous beta bursts with a duration of more than 100 milliseconds were retained (Tinkhauser et al., 2017). The following beta burst characteristics were calculated: rate, amplitude, and duration. Rate refers to the occurrence of the burst state normalized by time (in seconds), amplitude to the maximum value of the beta time-frequency amplitude during each burst occurrence, and duration to the time spent in the burst state. To prevent redundant detection of beta bursts across adjacent phases, a ‘flexible interval’ approach was implemented for segmentation. This approach allowed for dynamic adjustment of phase boundaries to accurately capture beta bursts that may have occurred at the transition between task phases. Initially, each phase was shortened by 0.2 seconds at both its start and end to create clear separation between phases. However, if a beta burst was detected at the boundary of a phase, the corresponding phase boundary was dynamically extended until the entire burst was captured. This methodology ensured that beta bursts were accurately assigned to the appropriate task phase and prevented artificial splitting of bursts that occurred during phase transitions, allowing for more precise analysis of the temporal distribution of beta bursts across different phases of the motor task. The left precentral and postcentral regions contralateral to the moving hand were chosen to visualize the results of aperiodic components and beta bursts, as these areas are most closely associated with motor function. Note that throughout this paper, ‘contralateral’ refers to these left hemisphere (dominant) regions opposite to the moving right hand, while ‘ipsilateral’ refers to regions in the right hemisphere (non-dominant) as the moving right hand.

The co-occurrence rate of bursts between motor regions and all the other regions were also calculated to examine the spatial distribution of beta bursts. The beta burst co-occurrence rate between brain regions A and B was defined as the proportion of time points with beta bursts in region A that also corresponded to beta bursts in region B. Specifically, it was calculated as the number of time points with beta bursts in both regions divided by the total number of time points with beta bursts in region A. Additionally, functional connectivity (FC) across all pairs of parcels was calculated using cross-correlation on the beta-band source-reconstructed EEG data. Cross-correlation was computed with time lags ranging between -100 and 100 ms (Christodoulakis et al., 2015), and the final FC matrix was constructed by selecting the maximum absolute correlation value across all time lags for each parcel pair. All FC values were normalized using Fisher-Z transformation.

### 2.8 Laguerre basis functions

First, the BOLD signal was up-sampled to 250 Hz to match the sampling rate of the EEG data. To ensure up-sampling did not introduce high-frequency artifacts, we analyzed the power spectra of BOLD signals before and after up-sampling (Supplementary Fig. S2), which showed no spurious high-frequency components that would compromise model estimation. The spherical Laguerre basis function expansion was used to estimate the HRF from EEG patterns. HRF fitting was performed separately for each subject and brain region. This was done by convolving the beta burst time-series with the HRF and using the least-squares criterion to minimize the prediction error of the BOLD signal (Prokopiou et al., 2022). Even in resting-state conditions with a notably low signal-to-noise ratio (SNR), this function can generate robust HRF estimates at the voxel level when using oscillatory EEG patterns (Leistedt & McEwen, 2012; Prokopiou et al., 2022). Here, a variant of the spherical Laguerre basis was applied to estimate BOLD signals based on the beta bursts in each region. The j-th spherical Laguerre basis function $b_{j}\left(n\right)$; j = 0,…, L-1; n = 1,…, M is given by

$$b_{j}\left(n\right)=\sqrt{\frac{j!}{\left(j+2\right)!}}\frac{e^{\frac{n}{2\alpha }}}{\sqrt{\alpha^{3}}}\cdot K_{j}\left(\frac{n}{\alpha }\right)$$ (1)

Where α is a parameter that determines the rate of exponential asymptotic decline of $b_{j}\left(n\right)$, and $K_{j}\left(n\right)$ is the j-th generalized Laguerre polynomial of order two, defined as

$$K_{j}\left(n\right)=\sum_{r=0}^{j}\left(\begin{array}{c} j+2\\ j-r \end{array}\right)\frac{{\left(-n\right)}^{r}}{r!}$$ (2)

The j-th spherical Laguerre basis function $b_{j}\left(n\right)$ was convolved with a Gaussian kernel G(μ,τ), with μ>0 and τ=1 . To avoid overfitting, especially when dealing with resting-state EEG-fMRI measurements characterized by a significantly lower SNR, the parameter L, representing the number of Laguerre basis functions, was varied between 1 to 3 and the parameter alpha, which controls the basis function rate to decay, was varied from 0.5 to 1. Besides, the range of the parameter mu, which controls the Laguerre time to peak, was varied between 2.5 and 5 (Prokopiou et al., 2022). An illustration of the effect of all parameters on the resulting dynamics is shown in Supplementary Figure S3. Threefold cross-validation was also used to assess the best estimation. Specifically, the task and resting state data were divided into three equal parts, the model was trained on two-thirds of the data, and its performance was evaluated on the remaining one-third in terms of the resulting correlation coefficient. This process was repeated three times, with each part of the data used once as the validation set. The unknown HRF curves were determined by least squares regression (LSR), which obtained the optimal estimate by minimizing the sum of squared residuals between the observed and predicted values. Finally, the predicted BOLD signal was calculated as the convolution of the estimated HRF curve with the beta bursts. To evaluate the model’s predictive performance, BOLD signals were estimated using beta burst matrices shuffled separately for each subject and brain region. The correlation coefficients obtained from surrogate data were compared to those obtained from empirical data. Additionally, all HRF curves were min-max normalized and averaged across subjects for visualization. The estimated HRF curves were characterized using three features: peak, area, and power. The HRF peak represents the maximum absolute amplitude of the curve, area refers to the total area under the HRF curve, and power was calculated as the sum of HRF squared values divided by curve length.

### 2.9 Receptor map processing

To investigate the biological basis of beta-burst HRF spatial patterns, their relationship with μ -opioid receptor (MU receptor) density, given its inhibitory role in motor control (Bezard et al., 2020; Wiskerke et al., 2012), was examined. A spatial correlation analysis was conducted between the receptor protein map and the HRF area map, which quantified the cumulative BOLD response to individual beta bursts. Only negative HRF area values were included in this analysis, as MU receptors are primarily inhibitory and would be expected to correlate with suppressive (negative) BOLD responses rather than positive activations. The receptor density map was assessed using Positron Emission Tomography (PET) tracer investigations. This dataset, recently made available by Hansen et al. (https://github.com/netneurolab/hansen_receptors; Hansen et al., 2022), consists of volumetric PET images aligned to the MNI-ICBM 152 nonlinear 2009 template. After alignment, participant-averaged images from each study were parcellated according to the DKT atlas.

### 2.10 Spatial correlation

To assess the spatial correlations between co-occurrence rates and FC patterns, as well as between HRF property patterns and MU receptor density, we employed the ‘spin test’—a null model that systematically disrupts associations between two topographic maps while preserving each map’s inherent spatial autocorrelation (Markello & Misic, 2021). FC and receptor maps were randomized and their association with co-occurrence rate and HRF area maps respectively were assessed. Spatial coordinates generated through this shuffling procedure were then used to create null models by performing a series of randomly sampled rotations. Node values were reassigned based on proximity to the nearest parcel after rotation (Hansen et al., 2022). This process was repeated 1000 times, with the 95^th^ percentile of shuffled occurrence frequencies in the spatial null models serving as the significance threshold.

### 2.11 Statistical analysis

For the statistical analysis, the non-parametric Wilcoxon signed-rank test was employed to compare behavioral data between left and right hands, and to compare beta burst characteristics and aperiodic components across different task phases and rest, as the data did not meet the assumption of normality (Woolson, 2005). To account for multiple comparisons, false discovery rate (FDR) correction was applied to all *p*-values obtained from the Wilcoxon signed-rank tests, controlling for the expected proportion of false positives. Given the relatively small sample size in this study, effect sizes (*r*) were calculated for all tests to ensure the reliability and interpretability of the findings (Fritz et al., 2012). Based on this criterion, effect sizes ≥0.5 were considered large effects, indicating strong statistical associations in our results (Coolican, 2017). Additionally, post hoc power analyses were conducted in R software using the MK Power package, which uses Monte Carlo simulations to estimate the empirical power of the statistical tests and determine the achieved statistical power for the observed results by using the mean and standard deviation of the paired differences (Kohl, 2020). The number of iterations was set to 5000 to ensure stable power estimation.

## 3 Results

### 3.1 Behavioral analysis

For the behavioral assessment, participants’ manual motor function was evaluated using the NHPT and BBT. Results revealed that participants completed the NHPT more quickly with their right hand (16.78 ± 1.82 seconds) compared to their left hand (18.57 ± 2.66 seconds), with a mean difference of -1.79 ± 1.77 seconds. The Wilcoxon signed-rank test showed that this difference was significant (left-right: *n* = 11, *z* = 2.67, *p* = 0.005, *r* = 0.80), indicating better right-hand performance in fine motor tasks. For the BBT, performance was relatively similar between the right hand (67.36 ± 5.50 blocks) and left hand (66.36 ± 5.43 blocks), with a small difference (1.00 ± 3.41 blocks) that did not reach statistical significance. Importantly, these values fall within the normative ranges established for healthy adults (Mathiowetz et al., 1985). These findings confirm that all participants exhibited normal motor function, which is crucial for the interpretation of the subsequent neurophysiological results.

### 3.2 Aperiodic components

**Fig. 2.**
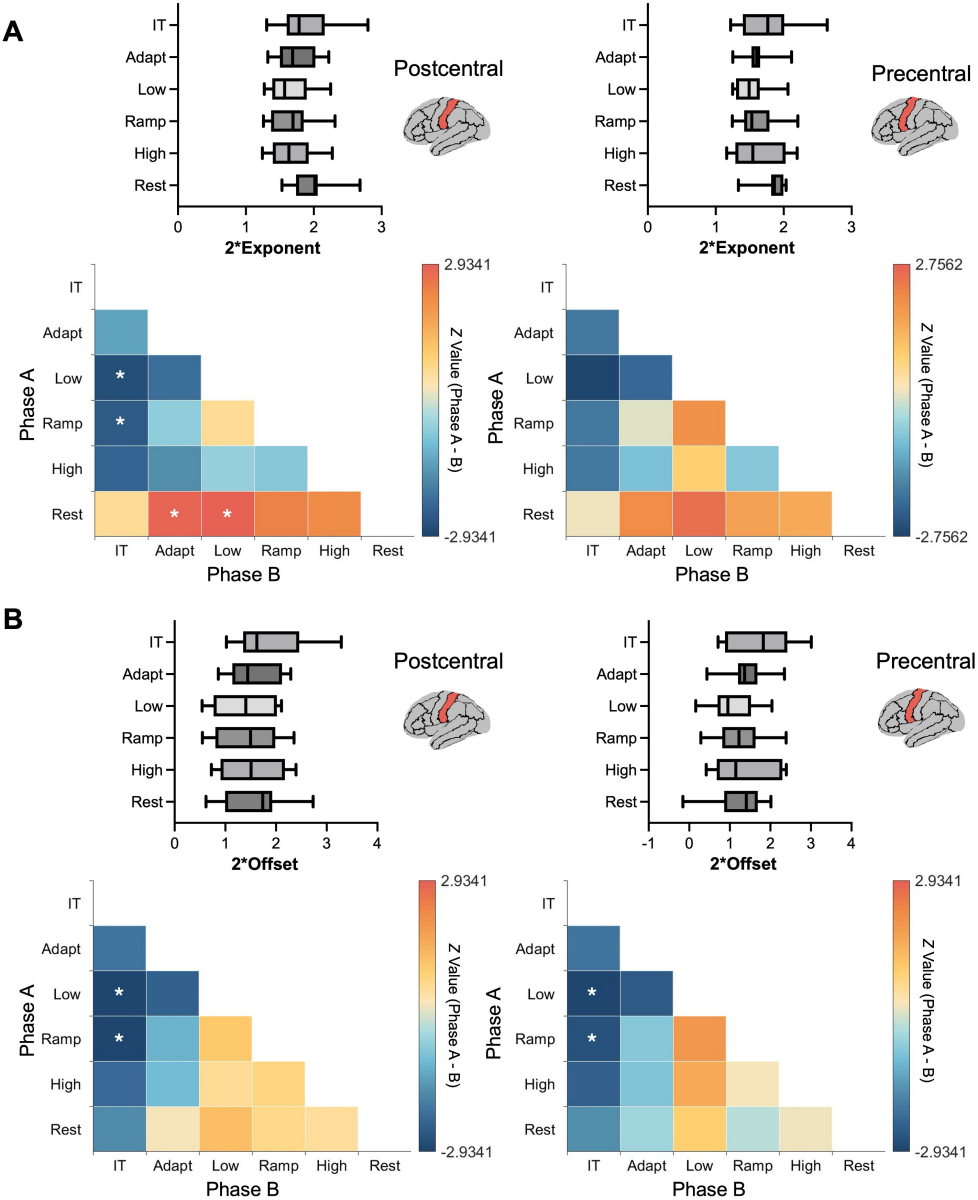
(A) Distribution and comparison of the aperiodic exponent in the left postcentral and precentral regions during the five task phases and the resting state (IT = inter-trial phase). (B) Distribution and comparison of the aperiodic offset in the left postcentral and precentral regions during the same task phases and rest. The heatmaps display *Z*-values representing pairwise comparisons between different phases of the motor task and rest (Y-axis vs. X-axis), with significant differences (*p* < 0.05, corrected using FDR) indicated by white asterisks (*). In the left postcentral region, the aperiodic exponent during rest was significantly higher than during the adaptation and low phases, while inter-trial phase showed a significantly higher exponent than the low and ramp phases. For the aperiodic offset, the inter-trial phase exhibited significantly higher values compared to the low and ramp phases in both precentral and postcentral regions.

Overall, the aperiodic exponent in the left pre- and post-central regions was higher during the inter-trial and resting states compared to the adaptation, low-force, ramp-force, and high-force phases (hereafter referred to as low, ramp, and high phases, respectively; Fig. 2A, upper panels). Specifically, in the left postcentral region (*n* = 11), the aperiodic exponent was significantly elevated during rest compared to the adaptation (*z* = 2.85, *p_fdr_* = 0.020, *r* = 0.86) and low (*z* = 2.93, *p_fdr_* = 0.020, *r* = 0.88), and during the inter-trial phase relative to the low (*z* = 2.76, *p_fdr_* = 0.020, *r* = 0.83) and ramp phases (*z* = 2.49, *p_fdr_* = 0.049, *r* = 0.75). In contrast, no significant differences were observed in the left precentral region across all the phases (Fig. 2A, lower panels).

The aperiodic offset in these regions was generally higher during the inter-trial and resting states than in the adaptation, low, ramp, and high phases (Fig. 2B, upper panels). Specifically, in the left postcentral region (*n* = 11), significant elevations in the aperiodic offset were observed during the inter-trial phase compared to the low phase (*z* = 2.93, *p_fdr_* = 0.009, *r* = 0.88) and ramp phases (*z* = 2.93, *p_fdr_* = 0.009, *r* = 0.88). In the left precentral region, the aperiodic offset was higher during the inter-trial phase compared to the low (*z* = 2.93, *p_fdr_* = 0.020, *r* = 0.88) and ramp phases (*z* = 2.67, *p_fdr_* = 0.049, *r* = 0.80; Fig. 2B, lower panels).

### 3.3 Beta burst temporal distribution

**Fig. 3.**
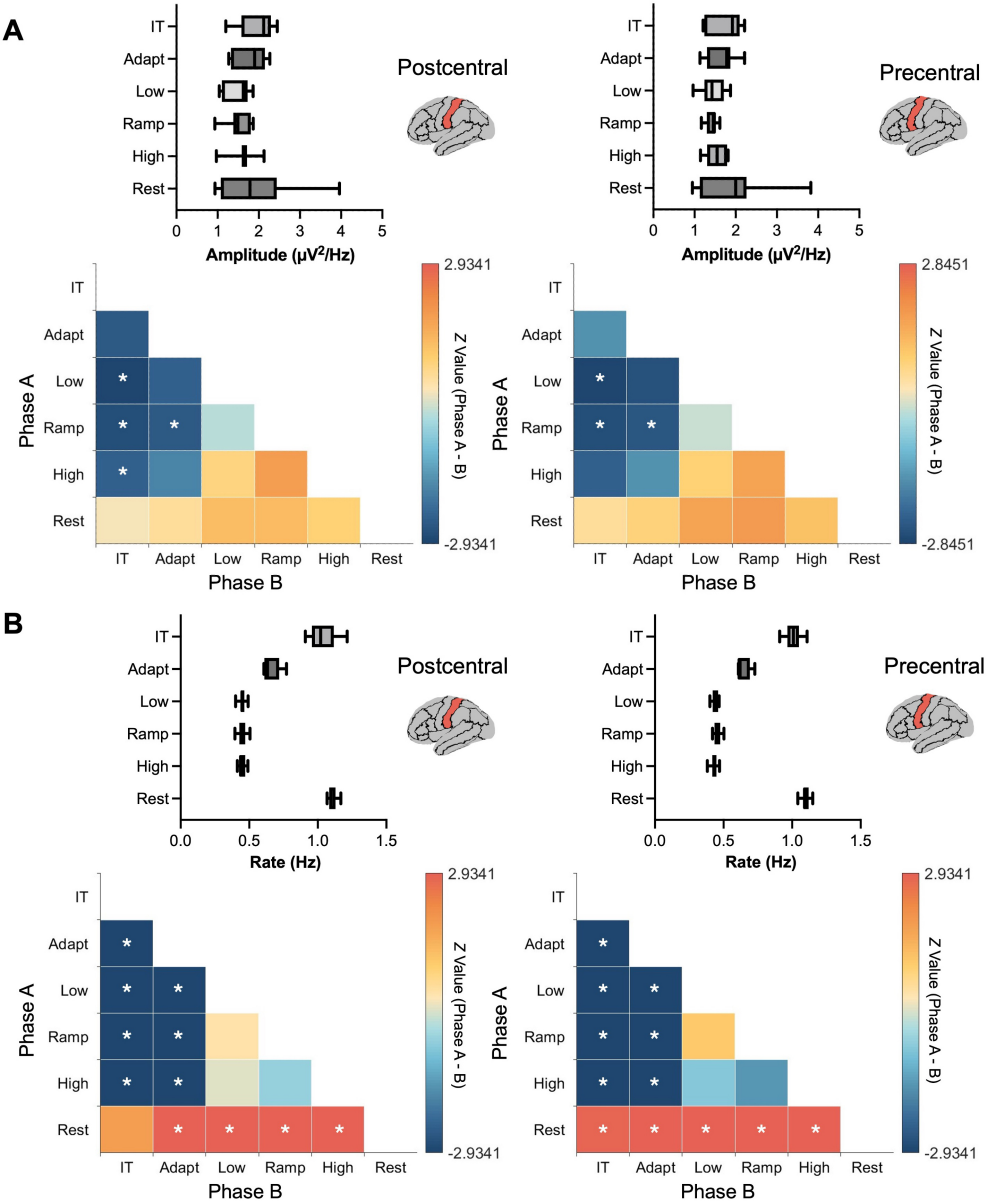
(A) Distribution and comparison of the beta burst amplitude in the left postcentral and precentral regions during the five task phases and the resting state (IT = inter-trial phase). (B) Distribution and comparison of the beta burst rate in the left postcentral and precentral regions during the same task phases and rest. The heatmaps display *Z*-values representing pairwise comparisons between different phases of the motor task and rest (Y-axis vs. X-axis), with significant differences (*p* < 0.05, corrected using FDR) indicated by white asterisks (*). In the left postcentral region, beta burst amplitude was significantly higher during the inter-trial phase compared to the low, ramp, and high phases, while the adaptation phase exhibited higher amplitude than the ramp phase. In the left precentral region, beta burst amplitude was greater during the inter-trial phase compared to during the low and ramp phases. For beta burst rate, the left postcentral region showed higher values during the inter-trial and resting phases compared to the adaptation, low, ramp, and high phases, and the adaptation phase exhibited a higher rate than the low, ramp, and high phases. Similarly, in the left precentral region, beta burst rate was elevated during the inter-trial and resting phases compared to the adaptation, low, ramp, and high phases, with the adaptation phase also showing a significantly higher rate than the low, ramp, and high phases.

Overall, beta burst amplitude in the left postcentral and precentral regions was higher during the inter-trial and resting phases compared to the adaptation, low, ramp, and high phases (Fig. 3A, upper panels). Specifically, in the left postcentral region (*n* = 11), beta burst amplitude was significantly elevated during the inter-trial phase compared to the low (*z* = 2.93, *p_fdr_* = 0.020, *r* = 0.88), ramp (*z* = 2.76, *p_fdr_* = 0.029, *r* = 0.83), and high phases (*z* = 2.40, *p_fdr_* = 0.034, *r* = 0.72), and during the adaptation phase relative to the ramp (*z* = 2.49, *p_fdr_* = 0.034, *r* = 0.75). In the left precentral region, beta burst amplitude was greater during the inter-trial phase compared to the low (*z* = 2.85, *p_fdr_* = 0.034, *r* = 0.86) and ramp phases (*z* = 2.67, *p_fdr_* = 0.034, *r* = 0.80), and the adaptation phase was characterized by a higher amplitude compared to the ramp phase (*z* = 2.49, *p_fdr_* = 0.040, *r* = 0.75) (Fig. 3A, lower panels).

Regarding beta burst rate, the left postcentral and precentral regions (*n* = 11) exhibited higher values during the inter-trial and resting phases than during the adaptation, low, ramp, and high phases (*z* > 2.84, *p_fdr_* < 0.01, *r* > 0.85 for all pairwise comparisons). Additionally, the adaptation phase exhibited a higher burst rate than the low, ramp, and high phases (*z* = 2.93, *p_fdr_* < 0.01, *r* = 0.88 for all pairwise comparisons; Fig. 3B, lower panels). Finally, the inter-trial and resting phases generally exhibited longer beta burst durations compared to the task execution phases. However, no significant differences were observed (Supplementary Fig. S4).

### 3.4 Beta burst spatial distribution

**Fig. 4.**
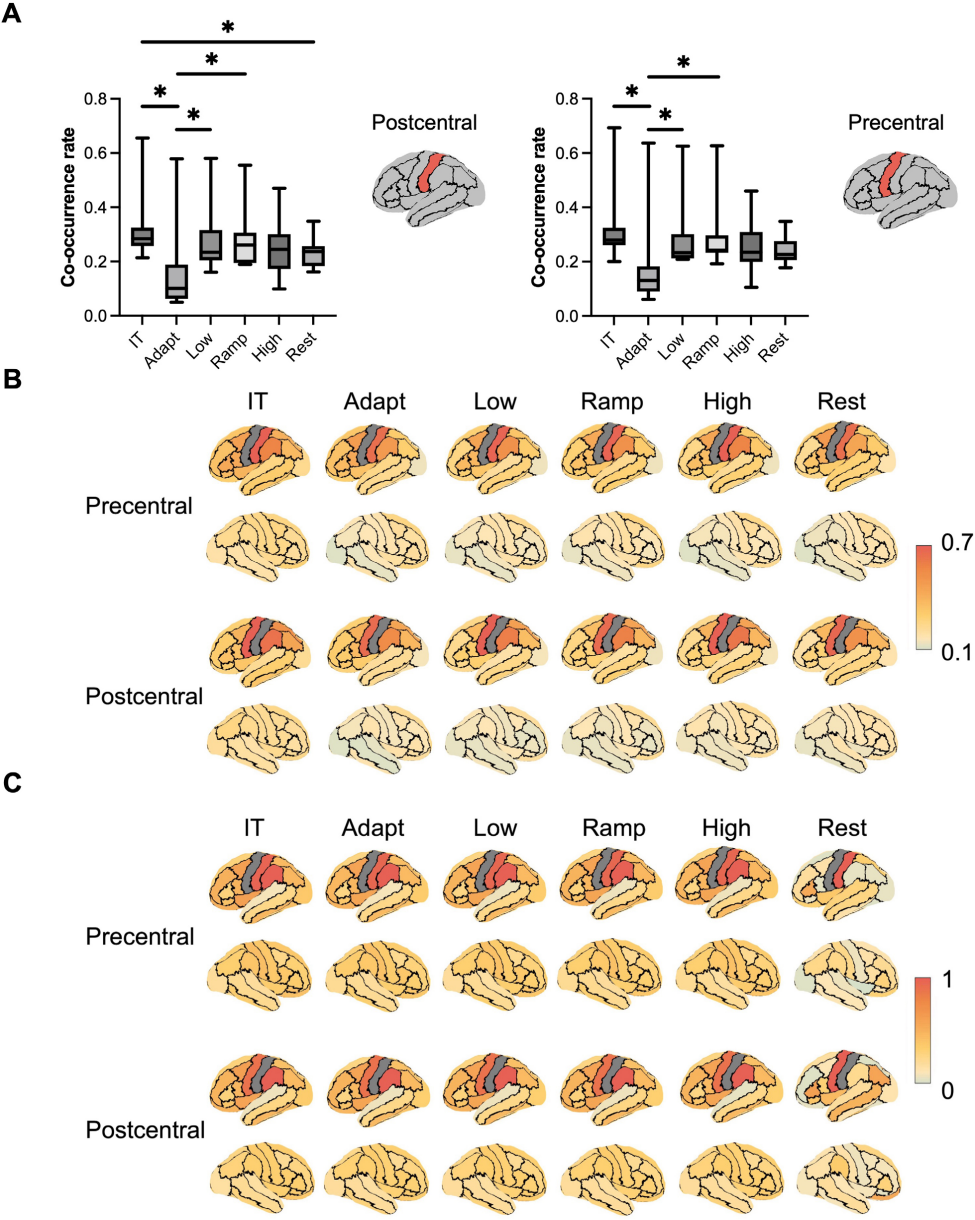
(A) Average co-occurrence rates between pre-/post-central regions and all other brain parcels during each task phase and rest. Overall, co-occurrence rates during the inter-trial phase were higher than other task phases and rest. Significant differences (*p* < 0.05, FDR-corrected) are indicated by asterisks (*). (B) Beta burst co-occurrence rates between the left precentral and postcentral regions and other regions during each task phase and the resting state. Across all task phases and rest, the highest inter-regional co-occurrence rates were observed between the precentral and postcentral regions. (C) Normalized absolute beta-band FC between the left precentral and postcentral regions and other regions during each task phase and the resting state. Across all task phases and rest, the strongest functional connectivity was observed between the precentral and postcentral regions.

The beta burst characteristics displayed similar values across all parcels, reflecting a relatively uniform distribution pattern during both the motor task and resting conditions (Supplementary Fig. S5). We aimed to investigate whether this uniformity was linked to patterns of beta burst co-occurrence across different regions, so we calculated the pairwise co-occurrence rate between each pair of regions. The co-occurrence rates were then averaged separately for each task phase and the resting state. In Figure 4A, the beta burst co-occurrence rate from the post-central region was higher during the inter-trial phase compared to the adaptation (*z* = 2.85, *p_fdr_* = 0.029, *r* = 0.86) and resting phases (*z* = 2.58, *p_fdr_* = 0.026, *r* = 0.78). The beta burst co-occurrence rate from the post-central region was lower during the adaptation phase compared to the low (*z* = -2.76, *p_fdr_* = 0.022, *r* = 0.83) and ramp phases (*z* = -2.67, *p_fdr_* = 0.024, *r* = 0.81). The beta burst co-occurrence rate from the pre-central region was higher during the inter-trial phase compared to the adaptation (*z* = 2.85, *p_fdr_* = 0.029, *r* = 0.86), and the co-occurrence rate during adaptation phase was lower than during the low (*z* = -2.67, *p_fdr_* = 0.037, *r* = 0.81) and ramp phases (*z* = -2.67, *p_fdr_* = 0.024, *r* = 0.81). These indicated enhanced synchrony of beta bursts during the inter-trial phase. Spearman correlation analysis of co-occurrence rate rankings across different phases from pre- and post-central regions revealed significant shifts between all pairwise phase comparisons (*p* < 0.0001 for all pairs), indicating that the spatial distribution patterns of beta burst synchrony were task-dependent rather than static across time. To examine the distinctiveness of motor regions compared to other regions, we extracted the co-occurrence rates between the left precentral and postcentral regions and all other regions across each task phase and the resting state (Fig. 4B). Our analysis revealed that the co-occurrence rates were highest between these two regions, across all task phases and the resting state. In contrast, co-occurrence rates in other regions were relatively low, except for the left supramarginal region. Additionally, co-occurrence rates in the left hemisphere were generally higher than those in the right hemisphere. To further validate these findings, we examined the absolute FC between the precentral and postcentral regions and all other brain regions (Fig. 4C). FC values were first converted to absolute values and then normalized by dividing all values by the maximum connectivity value to enable comparison across conditions. Consistent with the co-occurrence analysis, the strongest connectivity was observed between the precentral and postcentral regions themselves across all conditions. This convergent evidence from both beta burst co-occurrence and functional connectivity measures confirms the robust coupling within the primary sensorimotor network. This suggests that beta burst activity in contralateral motor-related regions predominantly overlapped with other contralateral motor regions, while showing low overlap with contralateral non-motor regions and ipsilateral regions. This pattern indicated a high degree of spatial specificity within motor-related networks during beta burst activity.

Given that FC patterns reflect aspects of neural synchronization, we examined the relationship between the absolute FC and the beta burst co-occurrence rate to better understand the neurophysiological basis of beta burst coordination. The correlations between FC and beta burst co-occurrence rates were calculated in motor-related regions (computed as the FC between motor-related regions and other regions), as well as the ones between their co-occurrence rates and the FC node strength of each brain region (computed as the mean FC across all connectivity with other regions; Table 1). Here, both FC and the co-occurrence rate for each region were averaged across subjects. The results show that for both the left precentral and postcentral regions, their average co-occurrence rates with all other regions were strongly correlated with their respective average FC values across all task phases and rest (*n* = 61). However, there was no significant correlation between these co-occurrence rates and the FC node strength values across all brain regions during any task phases and rest. These findings suggest that beta burst co-occurrence patterns may reflect network-specific synchronization mechanisms, particularly within motor-related regions, rather than being driven by beta-band connectivity properties.

**Table 1.** Correlation between co-occurrence rate (CR) and functional connectivity (FC) across brain regions during motor task phases and rest (r values).

	IT	Adapt	Low	Ramp	High	Rest
Precentral CR - Precentral FC	0.722*	0.682*	0.724*	0.697*	0.692*	0.462*
Postcentral CR - Postcentral FC	0.746*	0.776*	0.761*	0.753*	0.736*	0.456*
Precentral CR - FC node strength	-0.056	-0.077	-0.101	-0.106	-0.086	-0.168
Postcentral CR - FC node strength	-0.054	-0.059	-0.093	-0.079	-0.075	-0.091

Note: Analyses were performed in the left hemisphere for precentral and postcentral regions. “Precentral CR – Precentral FC” indicates the correlation between the CR of the precentral region with all other brain regions and its FC with those regions. “Precentral CR – FC node strength” indicates the correlation between the precentral region’s CR with all other regions and the node strength of those regions (mean FC of each region with all other regions). Similarly for the postcentral region.

* *p_spin_* < 0.001.

### 3.5 HRF estimation

To better understand the bandwidth of beta burst events and their relation to the BOLD signal bandwidth, we estimated their spectral content. As shown in Supplementary Figure S6, the original beta burst time-series in representative motor regions exhibited a pronounced peak below the fMRI Nyquist frequency (~0.24 Hz). Furthermore, the power spectra of the HRF-convolved signals across all subjects peaked consistently around 0.1 Hz, indicating that the HRF attenuates the high-frequency components and concentrates the spectral power well within the fMRI bandwidth. Therefore, even though the original burst time-series also contained spectral power above this frequency, there is still sufficient power within the bandwidth of the fMRI data (TR = 2120 ms) that can be reliably captured by BOLD measurements.

**Fig. 5.**
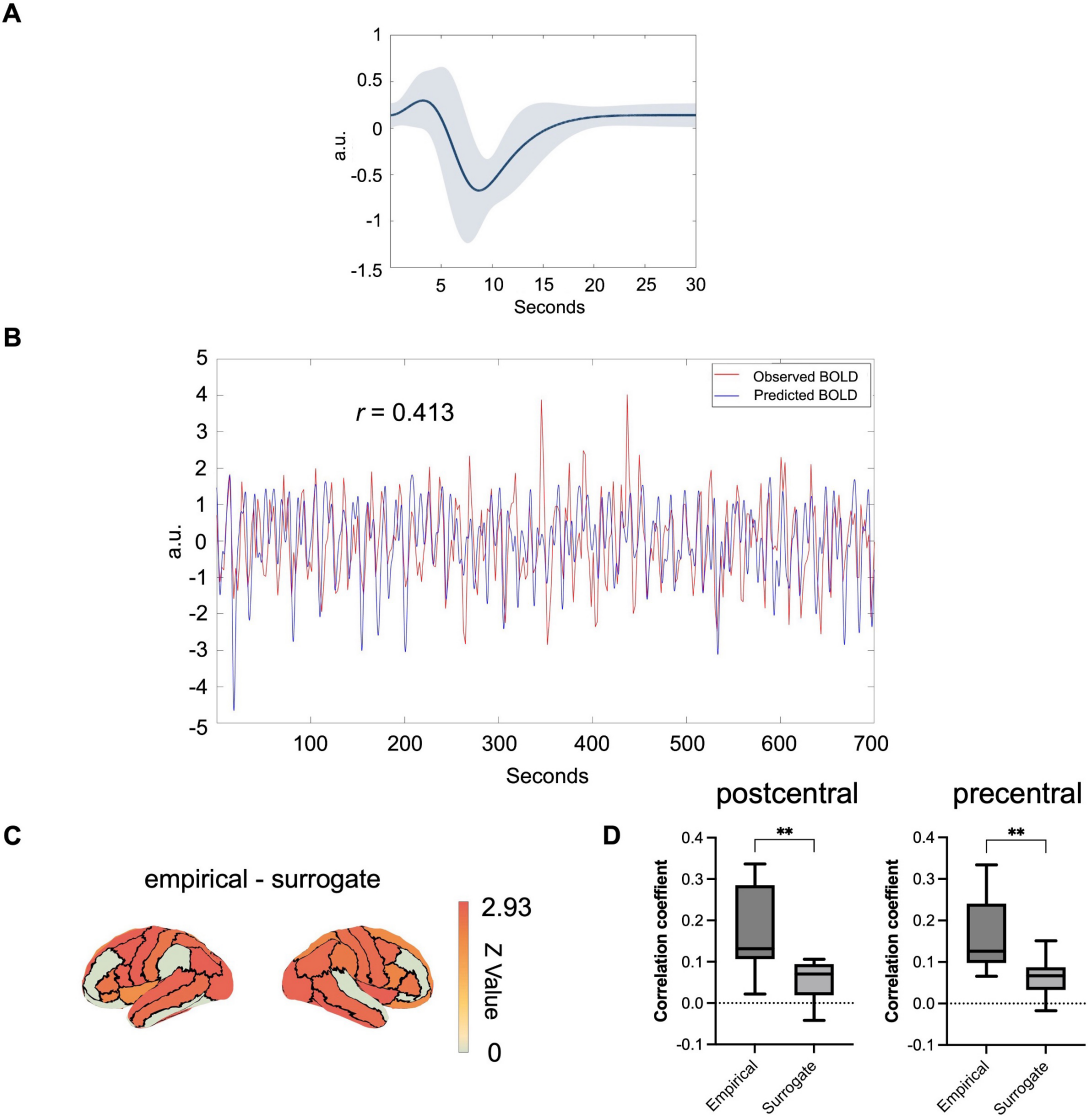
(A) Average normalized HRF curve in the right precentral region using the empirical data during the motor task. The blue curve corresponds to the mean HRF curve across all subjects. The blue shaded area corresponds to the standard error. (B) Comparison of predicted and observed BOLD signals from a representative subject in the right precentral region. The correlation coefficient between predicted and observed signals was *r* = 0.413. (C) Comparison of correlation coefficients between predicted and observed BOLD signals using original versus surrogate (shuffled) beta burst data across brain regions. The color map represents *z*-values obtained from the statistical comparison between empirical and surrogate conditions, with warmer colors (red-orange) indicating regions showing significant differences (*p* < 0.05, FDR-corrected). (D) Boxplots showing the distribution of correlation coefficients between predicted and observed BOLD signals across all subjects in the postcentral (left) and precentral (right) regions, for both empirical and surrogate beta burst data. Asterisks denote statistically significant differences between empirical and surrogate conditions (**p < 0.01).

The predictive performance of using spherical Laguerre functions was tested to estimate HRF dynamics between beta burst patterns and BOLD signals (Fig. 5). Figure 5A illustrates the group-averaged normalized HRF curve estimated using empirical beta bursts in the right precentral region during the motor task. An example of BOLD signal predictions from a representative subject in the same region is illustrated in Figure 5B, demonstrating robust correlation between the predicted and observed signals (*r_corr_* = 0.413, *p* < 0.0001). At the individual region level, the correlation coefficients between predicted BOLD signals from shuffled beta burst time-series and observed BOLD signals were significantly lower than those obtained using original beta burst patterns in the majority of brain regions (Wilcoxon signed-rank test, FDR-corrected; 52 out of 62 regions showed significant differences) (Fig. 5C), indicating that using the recorded beta bursts for prediction yields performance significantly above chance level. To further illustrate this difference at the group level, Figure 5D shows the distribution of correlation coefficients across all subjects in the pre- and postcentral regions, where empirical beta burst patterns yielded significantly higher correlations with the observed BOLD signals than surrogate data (postcentral: *z* = 2.58, *p* = 0.003, *r* = 0.78; precentral: *z* = 2.76, *p* = 0.002, *r* = 0.83; Wilcoxon signed-rank test). HRF estimation using surrogate beta bursts is also presented in Supplementary Figure S7. Overall, these results suggest that beta burst patterns can predict a fraction of the BOLD signal dynamics.

**Fig. 6.**
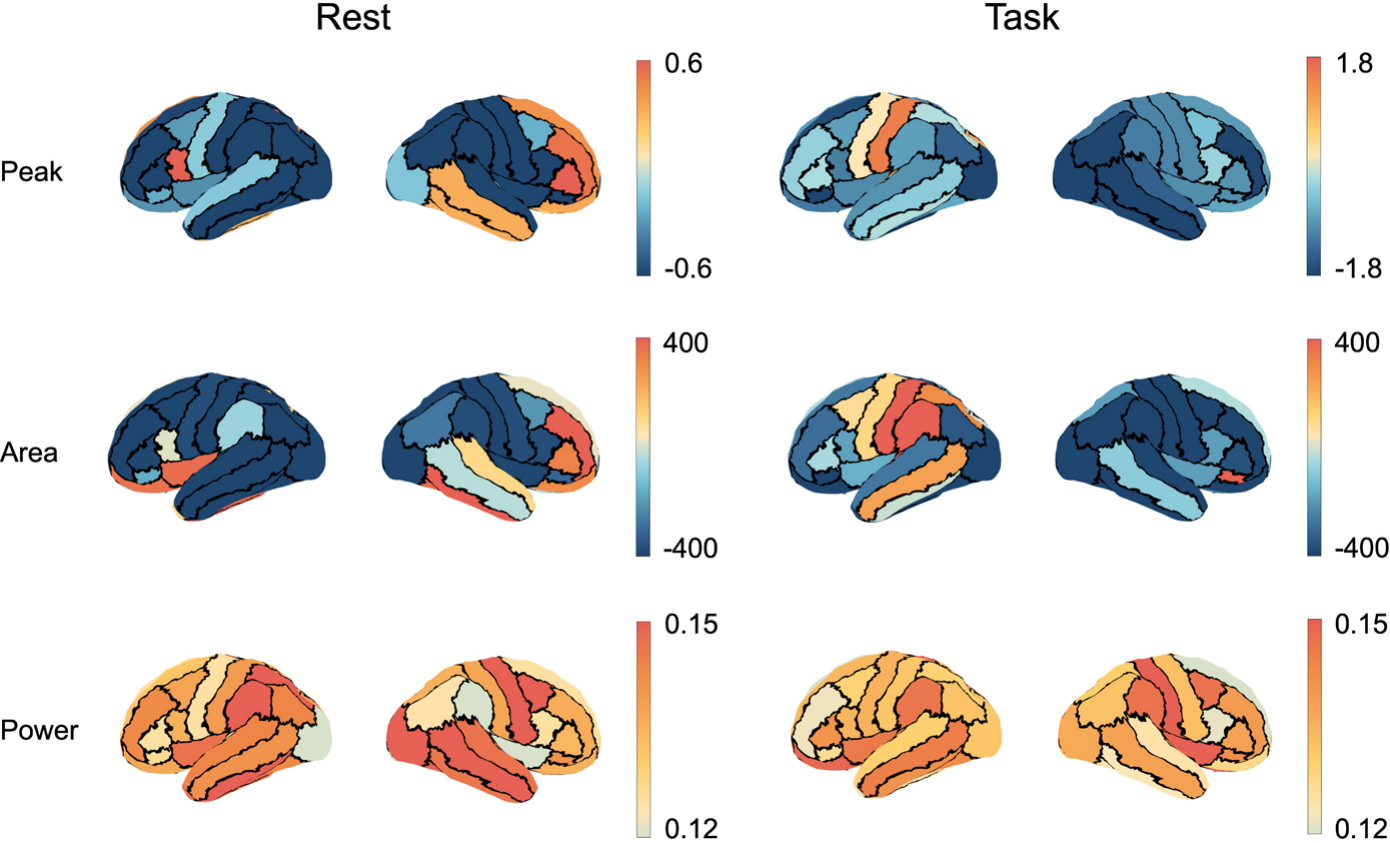
Group-level averaged maps of HRF curve peak, area, and power values. Upper panels: The resting-state HRF exhibited positive peaks in specific cortical regions, including the right frontal and temporal areas, while most regions exhibited negative HRF peaks. During the motor task, the HRF displayed widespread negative peaks across the cortical surface, with exceptions in the left postcentral and precentral areas. Middle panels: The resting-state HRF exhibited mostly negative area values, except in the left inferior frontal regions, and the right frontal and temporal regions. During the motor task, the HRF exhibited mostly negative areas in the right hemisphere, while the left hemisphere showed positive area values in the central and middle temporal regions. Lower panels: the HRF power during both rest and task states was similar across the entire brain.

With regards to the spatial patterns of HRF curves, examination of the peak (Fig. 6, upper panel) revealed that during rest, right frontal and temporal regions exhibited positive peaks, while other areas showed predominantly negative peaks. During the motor task, negative peaks were observed across most of the cortical surface, except in the left postcentral and precentral regions. The HRF curve area (Fig. 6, middle panel), representing the cumulative effect of a single beta burst on the BOLD signal, showed widespread negative areas during rest, except in the left inferior frontal and right frontal and temporal regions. The right hemisphere exhibited mostly negative area values, while positive areas were concentrated in the left hemisphere’s central and middle temporal regions. Although sensorimotor regions showed qualitative differences in both peak and area between rest and task conditions, statistical comparisons across subjects revealed no significant differences for either feature (Wilcoxon signed-rank test, *p* > 0.05). Additionally, we compared HRF curves over time in the contralateral motor regions between motor task and rest conditions (Supplementary Fig. S8). Paired T-test was performed at each time point, and we applied the Simes method for multiple testing correction to account for the large number of correlated statistical tests across time points (Loizides et al., 2015). This point-by-point comparison revealed no significant differences between the two conditions (*p* > 0.05, corrected for multiple comparisons). The HRF power (Fig. 6, lower panel) exhibited relatively uniform values across the cortical surface for both rest and task states, with minimal regional variation. These findings highlight the distinctive HRF characteristics in contralateral motor areas to the moving hand, where beta bursts elicited positive BOLD responses—as shown by both HRF peak and area values—exclusively in motor regions during movement.

**Fig. 7.**
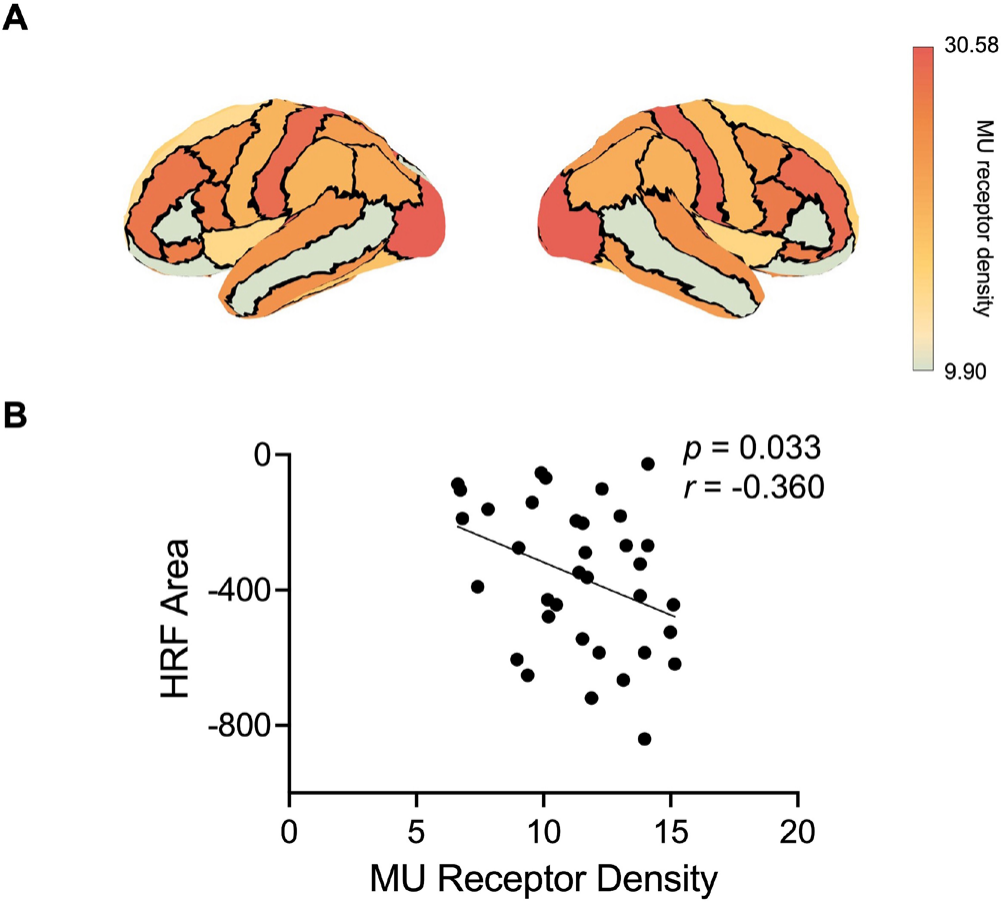
(A) MU (μ-opioid) receptor density map across each parcel. (B) Correlation between group-averaged negative HRF area values and MU receptor density values in the same regions. Negative HRF area values were negatively correlated with MU receptor density (*p* < 0.01 after the spin test).

As a first step towards elucidating the biological mechanisms underlying beta burst-induced HRF effects, the relationship between task-based group-averaged HRF area values and MU-receptor density (Fig. 7A) was examined. Focusing on regions with negative HRF areas—due to their predominance and the potential inverse relationship with MU-receptor inhibitory effects—a significant negative correlation between negative HRF area values and MU-receptor density was found (*n* = 35, *r_corr_* = -0.443, *p_spin_* < 0.01; Fig. 7B). Outlier analysis confirmed all data points were within Z-score < 3, indicating no statistical outliers.

### 3.6 Statistical power analysis

Post hoc power analysis revealed that the majority of significant pairwise comparisons described in sections 3.1–3.5 achieved adequate statistical power (> 0.85). However, some comparisons yielded lower power, including the left-right hand NHPT score difference (power = 0.78) in section 3.1, the aperiodic exponent comparisons for rest vs. ramp phase (power = 0.78) in section 3.2, and the beta burst amplitude comparison for adaptation vs. ramp phase in the precentral region (power = 0.76) in section 3.3. For these specific comparisons, the lack of statistical significance may reflect a lack in statistical power rather than a true absence of effects. These values, nevertheless, represent medium levels of statistical power as they indicate medium effect sizes.

## 4 Discussion

In this study, the spatiotemporal dynamics of beta bursts and aperiodic components were characterized during the different phases of a motor task and rest. A phase-specific modulation of burst characteristics and aperiodic components during the motor task was observed. A key finding was that beta bursts preferentially co-occurred between motor-related regions, a pattern that strongly aligned with areas of high FC. Beta bursts were also found to be correlated to dynamic changes in BOLD activity. Specifically, motor areas contralateral to the moving hand exhibited positive BOLD responses during task execution but negative responses during rest, whereas other brain regions exhibited negative BOLD responses during both conditions. To our knowledge, these results are the first systematic investigation of how transient EEG events, particularly beta bursts, influence BOLD activity during different motor states. Below, we further discuss the implication of these findings for understanding both the spatiotemporal organization of beta bursts and their relationship with hemodynamic responses in the context of motor control.

### 4.1 Aperiodic activity

Aperiodic components of neural signals, specifically the exponent and offset, serve as indicators of excitation-inhibition (E-I) balance, with reduced values suggesting E-I dysregulation (Gao et al., 2017; Lombardi et al., 2017; Wang et al., 2022). Elevated beta-band aperiodic exponent and offset values were observed in motor-related regions during rest and inter-trial phases compared to intra-movement phases (adaptation, low, ramp, and high force), confirming the task-dependent modulation of aperiodic activity (Monchy et al., 2024). This dynamic pattern suggests a sophisticated regulatory mechanism: during movement initiation and execution, motor cortical circuits exhibit increased excitatory neural activity to facilitate motor output, reflected in a shift toward excitation in the E-I balance. Subsequently, upon movement completion, neural systems actively restore E-I homeostasis, presumably to prevent prolonged hyperexcitability and suppress unwanted motor activation. This bidirectional modulation highlights the nervous system’s capacity to dynamically regulate cortical excitability according to motor demands while maintaining stable neural function during rest.

### 4.2 Beta burst characteristics

Beta burst amplitude has been shown to correlate with motor performance adaptation (Torrecillos et al., 2018). In Parkinson’s disease, beta bursts with higher amplitude were associated with greater motor impairment during the motor task, whereas bursts with lower amplitude corresponded to better motor performance (Tinkhauser et al., 2017). Our findings indicate that beta burst amplitude was higher during the inter-trial phases compared to the intra-movement phases, particularly during the low and ramp phases. This temporal pattern aligns with the established framework of beta event-related desynchronization and synchronization (ERD/ERS), characterized by substantial beta power attenuation during movement preparation and execution, followed by post-movement rebound. These dynamic patterns reflect the orchestrated excitation and inhibition within motor networks (Solis-Escalante et al., 2012). They also suggest a sophisticated control mechanism: beta burst amplitude functions as a dynamic gate, suppressing unnecessary motor activity while selectively facilitating task-relevant movements. This interpretation bridges clinical observations with fundamental motor control principles, suggesting that precise regulation of beta burst amplitude is crucial for optimal motor performance.

Beta bursts have emerged as critical markers of motor inhibition, functioning as spatiotemporal filters that regulate neural information flow through transient functional inhibition (Lundqvist et al., 2024). These intermittent events are not confined to specific cognitive processes, but manifest across diverse neural states, including working memory, motor tasks, and resting conditions (Lundqvist et al., 2016). In the motor cortex, the occurrence of beta bursts is associated with motor inhibition and decreased movement velocity (Khanna & Carmena, 2017). During the task, the brain must continuously balance excitatory and inhibitory operations, resulting in a significant reduction in this inhibitory control. Our results suggest that the beta burst rate of the motor cortices (precentral and postcentral regions) during the motor task (adaptation, low, ramp and high-level phases) was significantly lower than that in the inter-trial and resting state, consistent with findings from previous findings (Brady et al., 2020). This temporal pattern suggests enhanced motor function suppression during non-movement phases, reflecting a sophisticated control mechanism that modulates motor output according to task demands.

### 4.3 Beta burst co-occurrence rate

Examination of burst co-occurrence patterns from the pre- and post-central regions revealed distinct temporal and spatial organization: co-occurrence rates peaked during the inter-trial phase, reached their minimum during adapt phase, and showed consistently elevated values between motor regions. The lowest co-occurrence rate during the adaptation phase likely reflects a transient decoupling of the motor cortex from distributed brain networks, as local sensorimotor circuits are preferentially engaged in establishing the appropriate motor output during this initial transition from rest to active force production. Pre-movement beta oscillations in the motor cortex have been shown to be significantly reduced during early adaptation, reflecting an increased adaptive drive as the motor system recalibrates to new task demands (Darch et al., 2020). Furthermore, beta frequency oscillations have been proposed to support synchronization over long cortical distances, and a reduction in beta-mediated interregional coupling during motor transitions may indicate a shift from global network coordination to local cortical processing (Engel & Fries, 2010; Kopell et al., 2000). It should be noted, however, that the high co-occurrence between adjacent sensorimotor areas may be partially influenced by volume conduction effects. Nevertheless, pairwise co-occurrence rates between motor and other cortical areas strongly correlated with functional connectivity patterns, suggesting that beta burst co-occurrence may serve as an indicator of inter-regional communication strength and underlying neural connectivity (van den Heuvel & Hulshoff Pol, 2010). Previous studies have demonstrated that beta burst activity orchestrates movement through widespread influence across motor circuits, encompassing both cortical and subcortical structures (Cagnan et al., 2019; Yu et al., 2021). Our findings extend this concept and suggest that elevated co-occurrence during movement transitions reflects the recruitment of extensive neural networks for synchronized inhibitory control (Pfurtscheller & Lopes da Silva, 1999). Conversely, the reduced co-occurrence observed during active movement likely represents a shift toward selective engagement of task-relevant regions, with decreased synchronization in non-motor areas (Wheaton et al., 2005). The low co-occurrence rate observed in the resting state likely represents a baseline level (Chen & Hallett, 1999), characterized by independent regional operation with limited coordinated inhibitory control. These patterns reveal how the brain precisely modulates beta burst synchrony according to motor demands: employing heightened cross-regional coordination during movement transitions, selective engagement during execution, and low-level synchronization during rest. Such systematic modulation appears critical for optimizing motor control through precise spatiotemporal coordination of neural activity.

### 4.4 HRF properties

To examine the dynamic effect of beta burst patterns on local hemodynamic signals, basis expansions using spherical Laguerre functions were used for estimating the HRF and predicting BOLD signal fluctuations (Leistedt & McEwen, 2012; Prokopiou et al., 2022). During the motor task, the HRF peak and area were found to be positive in the contralateral precentral and postcentral regions, including M1, premotor, and SMA. This task-specific positive coupling between beta bursts and BOLD activity in contralateral motor regions, which contrasts with the negative coupling observed during rest, suggests a potentially distinct modulatory role of beta bursts across different brain states (Feingold et al., 2015; Torrecillos et al., 2018; Wessel, 2020). In contrast, during the resting state, the HRF peak and area in these contralateral motor regions were found to be negative. However, it should be noted that the differences in HRF features between task and rest conditions did not reach statistical significance, which may be due to the limited sample size (n = 11). Furthermore, in most other brain regions, the HRF peak and area remained negative regardless of whether participants were at rest or engaged in the motor task. This finding aligns with previous observations of positive HRF peaks between EEG power and BOLD signals in corresponding regions during motor tasks (Prokopiou et al., 2022). Using a larger dataset (78 total participants, 30 healthy controls), the general linear model (GLM) analysis (Briley et al., 2021) also revealed positive coupling between beta bursts and BOLD signals in motor regions during task performance, consistent with our findings, although their results suggested activation patterns during both task and rest. This difference might be attributed to block design approach used that did not allow for separate characterization of task and rest-state effects, and their use of canonical HRF which might overlook potential variations in the temporal dynamics of neurovascular coupling across different brain states (Handwerker et al., 2004; Lindquist et al., 2009).

Interestingly, only the contralateral motor regions exhibited positive responses during the task, whereas the ipsilateral motor regions consistently showed negative responses during both task and resting states. This pattern may reflect the competitive interactions between hemispheres during unimanual tasks, where the contralateral hemisphere’s activation is dominant while the ipsilateral hemisphere maintains an inhibitory state to balance motor control (Waters et al., 2017). This observation aligns with findings demonstrating an intrinsic balance of excitatory and inhibitory couplings within the motor network, whereby during unimanual movements, connectivity toward the contralateral primary motor cortex is enhanced while neural coupling toward ipsilateral motor areas is reduced through transcallosal inhibition and top-down modulation (Grefkes et al., 2008). This dynamic interplay between facilitation and inhibition within and across hemispheres suggests a finely tuned regulatory mechanism that optimizes motor control for specific task demands.

This distinct pattern of hemodynamic responses in contralateral motor regions—positive during tasks and negative at rest—reveals a potential sophisticated dual role of beta bursts in motor control. While beta bursts are traditionally viewed as inhibitory signals (Jenkinson & Brown, 2011), our findings suggest a more nuanced mechanism. During motor tasks, beta bursts may facilitate movement through “selective inhibition”—a process that enhances motor precision by suppressing non-essential neural activity while allowing task-relevant activation to emerge (Engel et al., 2001; Feingold et al., 2015). This modulation may enhance neural activation, leading to a positive HRF response linked to precise motor control. In contrast, the negative HRF response during rest likely reflects a different functional state where beta bursts maintain neural “idling” - actively suppressing spontaneous motor activity while preserving the capacity for rapid response to potential movement demands (Shmuel et al., 2006). Additionally, a higher frequency of beta bursts was observed during rest compared to the motor task, which may contribute to neural circuits becoming fixed in a particular state, potentially resulting in freezing or sustained states with reduced neural activation (Feingold et al., 2015). These findings suggest that beta bursts may dynamically modulate the excitatory-inhibitory balance within motor regions, facilitating motor control during tasks while preserving a low-activation state during rest. Future studies with larger sample sizes are warranted to further determine whether the observed directional differences in hemodynamic responses between task and rest conditions reflect a statistically robust state-dependent modulation of neurovascular coupling by beta bursts.

### 4.5 Relationship with MU receptor

A negative correlation was found between negative HRF areas and MU-opioid receptor density. MU receptors, which exert inhibitory control over motor pathways, play a crucial role in both ameliorating Parkinsonian motor symptoms and suppressing excessive motor impulses (Bezard et al., 2020; Wiskerke et al., 2012). This correlation suggests a potential mechanistic link: the magnitude of beta burst-induced negative hemodynamic responses appears to be modulated by regional MU receptor density, indicating that these inhibitory receptors may contribute to the neural-hemodynamic coupling that underlies motor control. This finding provides novel insights into the relationship between neurotransmitter systems and hemodynamic responses, potentially offering new perspectives on therapeutic approaches for motor disorders. However, it should be noted that the receptor employed density maps were derived from a separate cohort and may not fully represent the individual variability present in our sample, and future studies incorporating subject-specific receptor imaging would strengthen these interpretations.

## 5 Limitations

Our study utilized a relatively small sample size of 11 participants. However, the calculated effect sizes and post hoc power suggest that in most cases (more than 90% for the examined measures), the achieved effect size and power values were above 0.8 and 0.9 respectively, which are considered as robust effects in the literature. In some cases, the resulting power values were below 0.8 (e.g. 0.75), which suggests that these results should be treated with caution. While larger cohorts could potentially enhance the generalizability of the results, our sample was adequate to reveal meaningful trends and validate our approach.

While a comprehensive examination of both beta bursts and aperiodic components was performed, the aperiodic components’ influence on BOLD signals was not directly investigated. Given that both aperiodic metrics (exponent and offset) and beta bursts are associated with motor inhibition, they may generate similar BOLD responses. Future investigations should explore the hemodynamic effects of aperiodic components to provide a more complete understanding of motor-related brain activity. Such research could delineate the distinct and overlapping contributions of transient and aperiodic neural events to hemodynamic responses, potentially revealing new insights into motor control mechanisms.

## 6 Conclusions

The spatiotemporal distribution of beta aperiodic components and beta burst characteristics were examined during a motor task and at rest. We showed that both aperiodic components and beta burst characteristics were significantly elevated in the inter-trial phase compared to other task execution phases. Additionally, the distinct impact of beta bursts on the BOLD response was highlighted. Specifically, beta bursts in the contralateral motor regions produced positive HRF responses during motor tasks, whereas negative responses were observed during rest. In contrast, other brain regions consistently exhibited negative HRF responses in both task and resting states. These findings advance our understanding of the relationship between hemodynamic signals and transient neurophysiological events, particularly beta bursts, as captured through simultaneous EEG-fMRI recordings. This knowledge may also contribute to more accurate interpretation of neuroimaging data in clinical assessments and could inform the development of refined monitoring tools for sensorimotor function.

## Supplementary Material

Supplementary Material

## Data Availability

The data supporting this study’s findings are not openly available due to reasons of sensitivity and are available from the corresponding author upon reasonable request.
