# Supplementary material for: Beta bursts spatiotemporal profiles and their links to hemodynamic responses during movement and rest
Source: Imaging Neurosci (Camb). 2026 May 27;4:IMAG.a.1255. doi: 10.1162/IMAG.a.1255 (PMC13218349; doi:10.1162/IMAG.a.1255)
Supplement: Supplementary Material [file IMAG.a.1255_supp.pdf]

Supplementary Materials

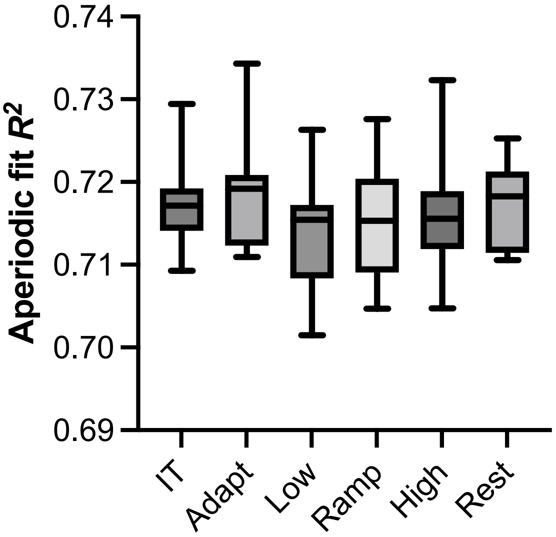

Fig. S1. Distribution of R^2 values between observed and fitted aperiodic power spectra across the five task phases and resting state (each boxplot represents 11 subjects averaged across brain regions). The consistently high R^2 values (>0.71) across all conditions demonstrate reliable aperiodic parameter estimation with no significant differences between phases, validating the accuracy of the spectral parameterization approach.

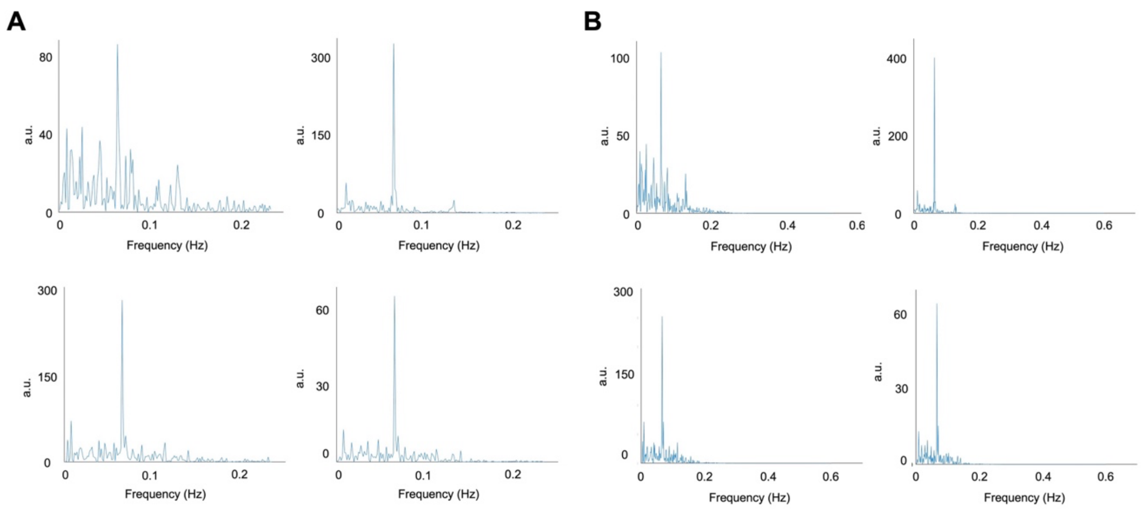

Fig. S2. (**A**) Power spectrum of four example original BOLD signals. (**B**) Power spectrum of the same four BOLD signals after up-sampling to 250 Hz. Each plot in (A) corresponds to the plot directly below it in (B), showing the same signal before and after up-sampling. The power spectra demonstrate that up-sampling does not introduce spurious high-frequency components that could affect HRF model estimation.

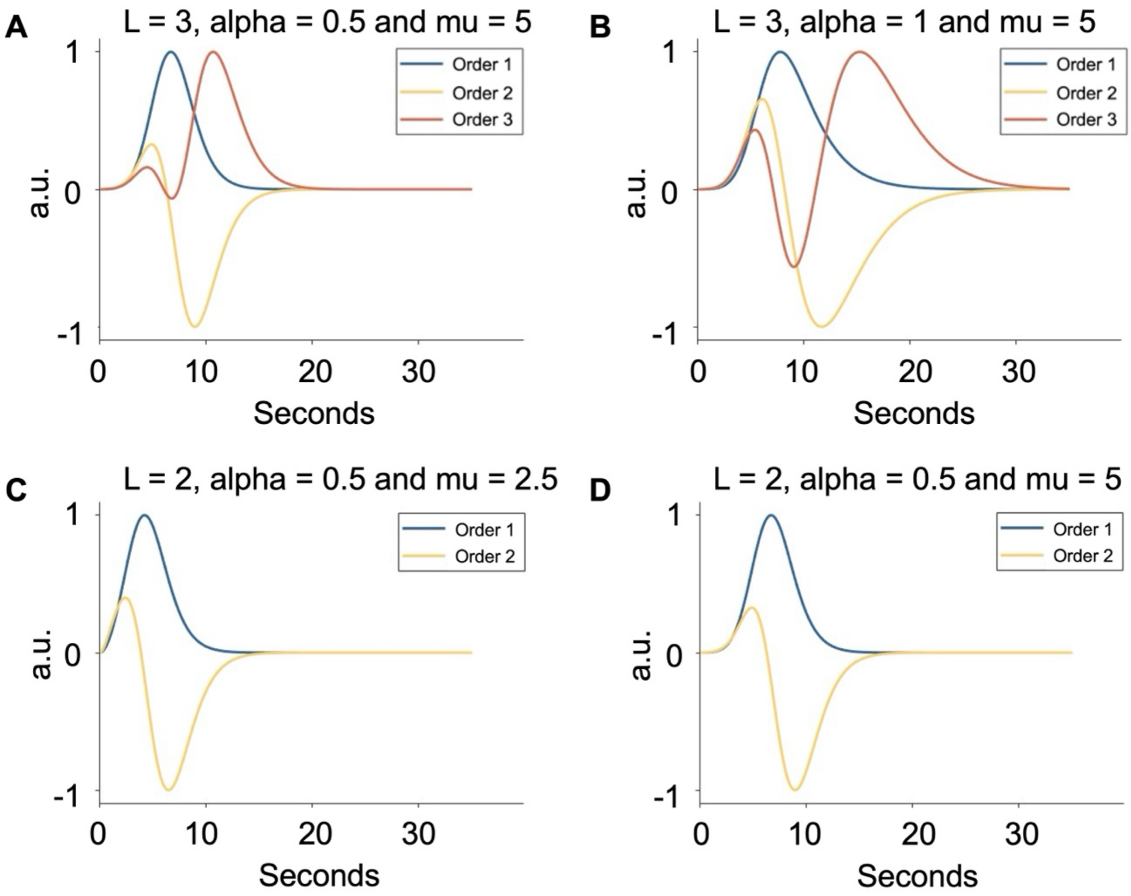

Fig. S3. Illustration of spherical Laguerre basis functions (SLBF) for different values of L, alpha, and mu. Here, L denotes the model order, representing the total number of spherical Laguerre functions; alpha is the rate of exponential decay, and mu controls the time delay relative to the input onset (t=0). (A-B) SLBFs of orders 1-3 with fast (alpha=0.5) and slow (alpha=1) dynamics. (C-D) SLBFs of orders 1-2 with either an instantaneous (mu=2.5) or delayed (mu=5) response.

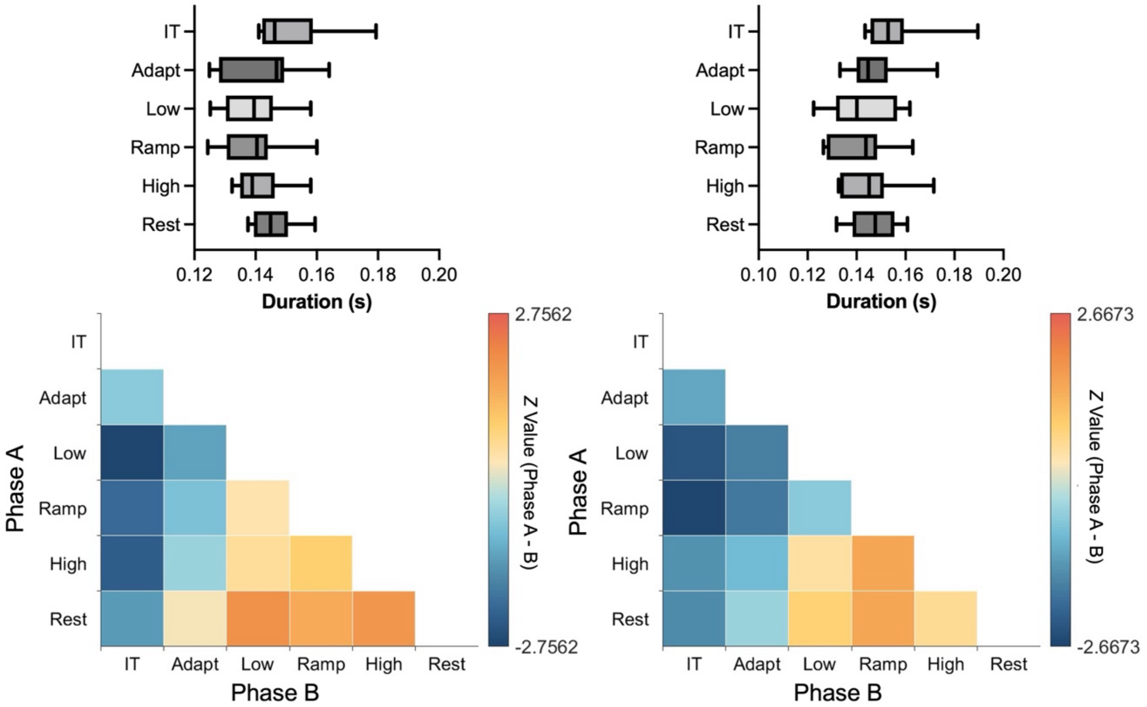

Fig. S4. Distribution and comparison of beta burst duration in the left postcentral and precentral regions across the five task phases and the resting state. The heatmaps display Z-values representing pairwise comparisons between different phases of the motor task and rest (Y-axis vs. X-axis). No significant differences between phases were found.

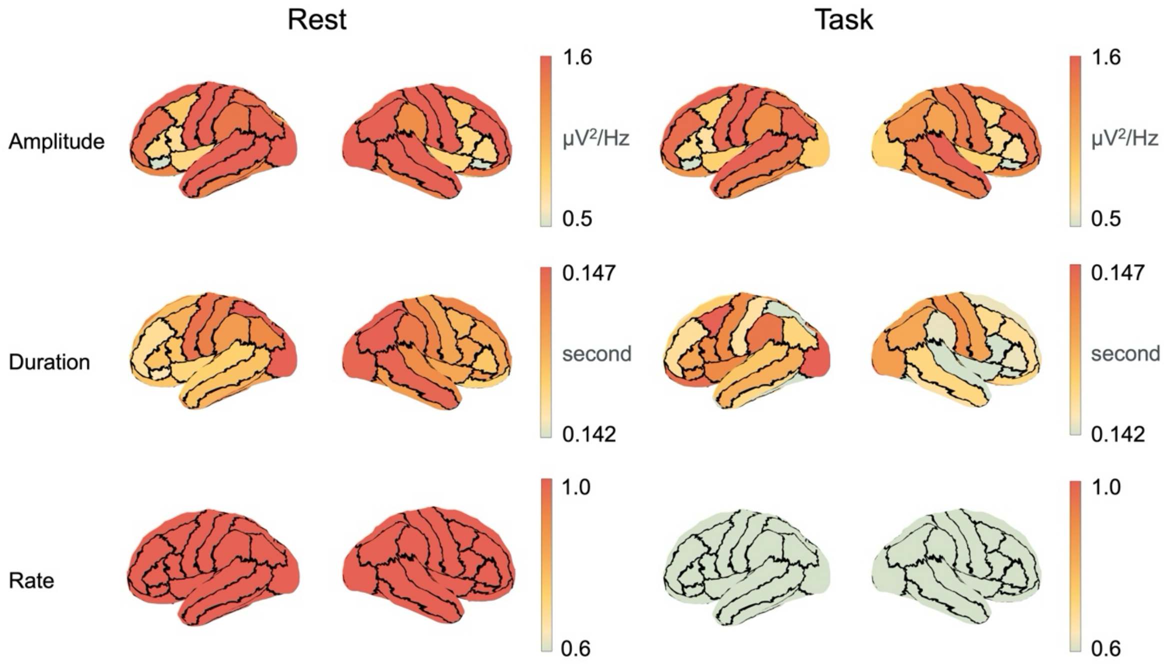

Fig. S5. Spatial distribution of beta burst characteristics across each parcel during the motor task and resting state. The amplitude, duration, and rate of beta bursts exhibited similar values across all parcels for both the resting and motor task conditions (no significant differences observed).

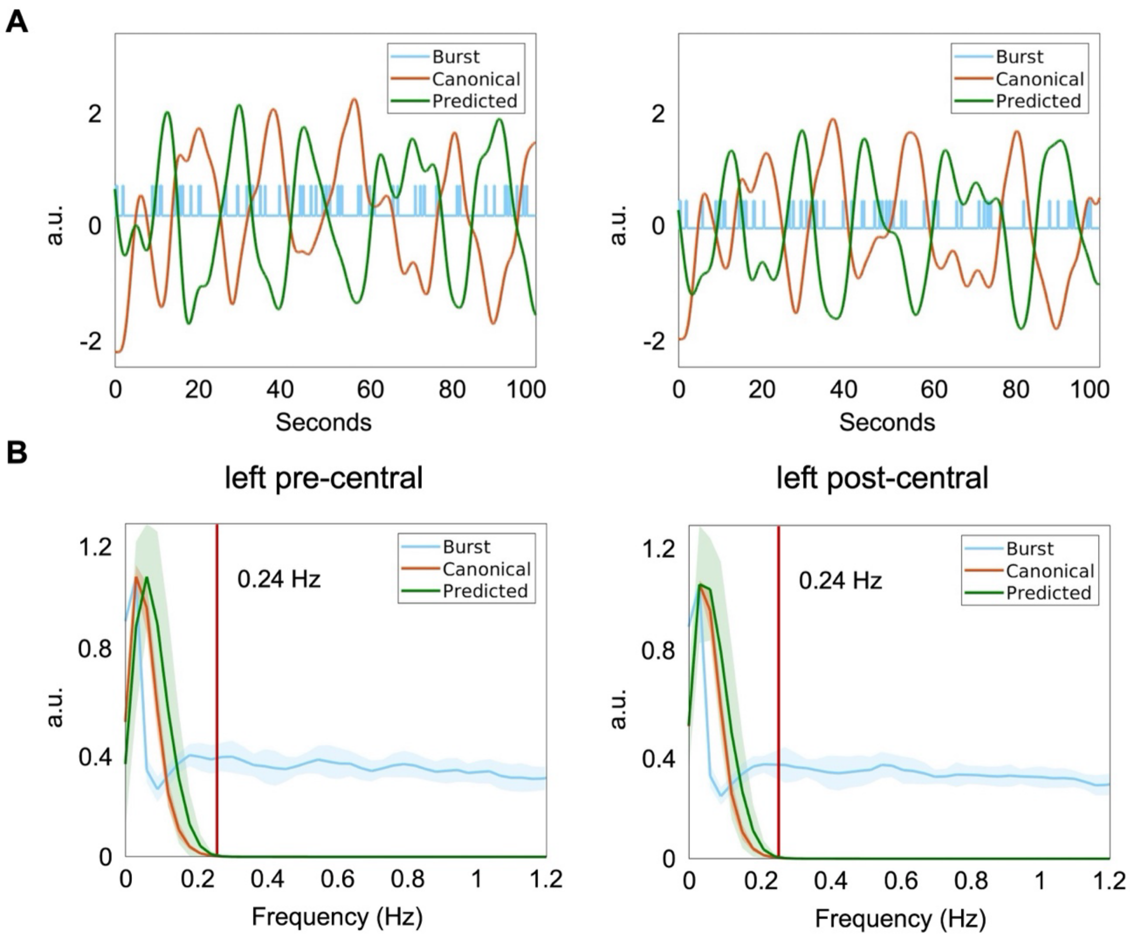

Fig. S6. Beta burst time-series, HRF-convolved signals, and their power spectra in the left pre- and postcentral regions. **(A)** A representative 100-second segment of task-state data from a single participant showing time-series from the left precentral (left) and left postcentral (right) regions. The light blue trace shows the original beta burst time-series (binary events), the orange trace shows the burst time-series convolved with the canonical HRF, and the green trace shows the burst time-series convolved with the HRF estimated using spherical Laguerre basis functions. All traces were *z*-scored to enable visual comparison. A 100-second window was selected for visualization clarity, as individual burst events become harder to distinguish in longer recordings. **(B)** Group-level normalized power spectra computed from the full-length task-state data for the left precentral (left) and left postcentral (right) regions. Solid lines represent the mean across all subjects and shaded areas indicate ± standard deviation. Power spectra were normalized to their respective maximum values to facilitate visual comparison of spectral shapes. Vertical red lines indicate the Nyquist frequency (~0.24 Hz) of the fMRI data (TR = 2120 ms).

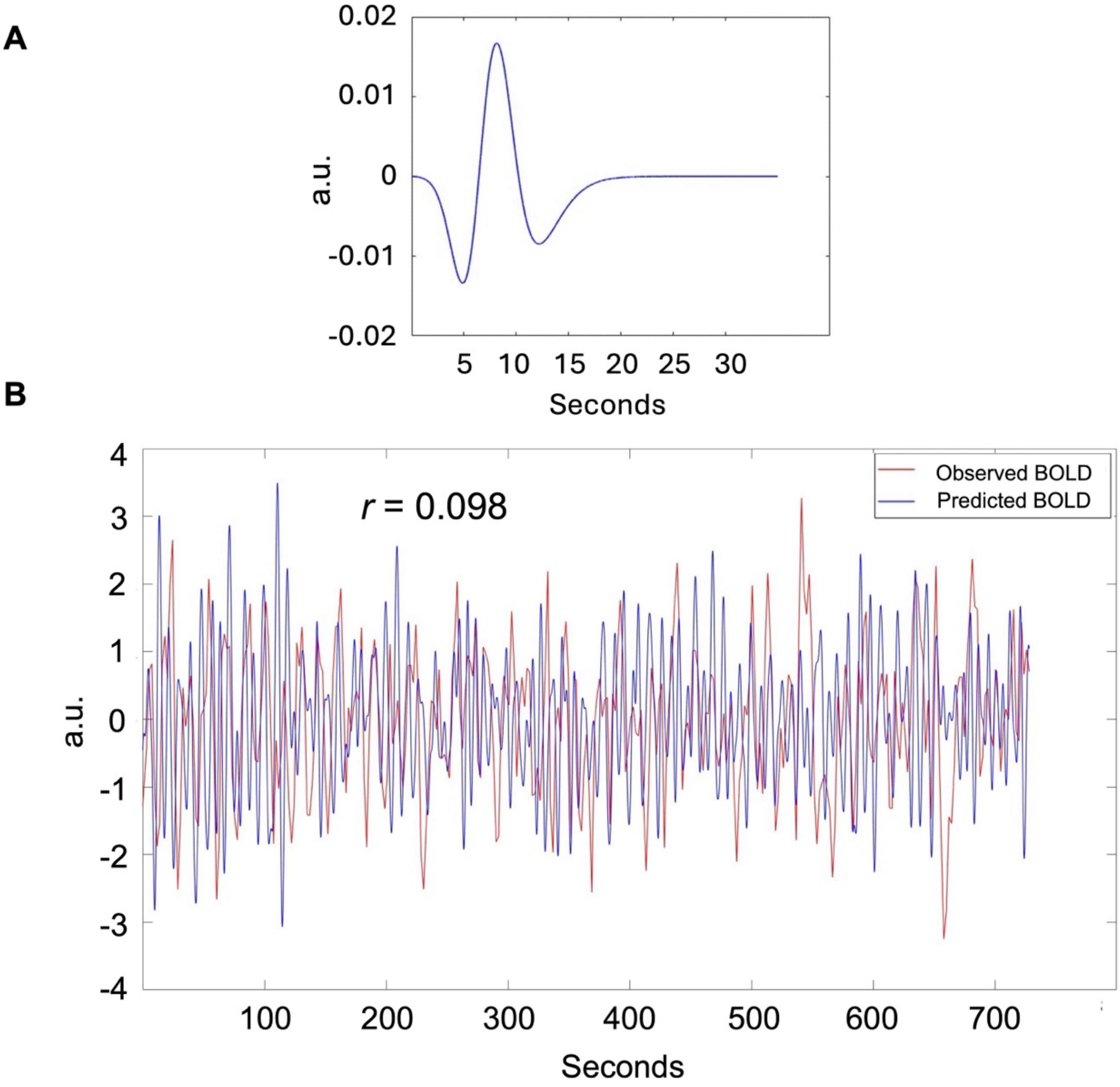

Fig. S7. (A) An example of an estimated HRF curve using the surrogate data during the motor task. **(B)** Comparison of predicted and observed BOLD signals corresponding to the same HRF estimation as shown in (A), with a correlation coefficient $r = 0.098$.

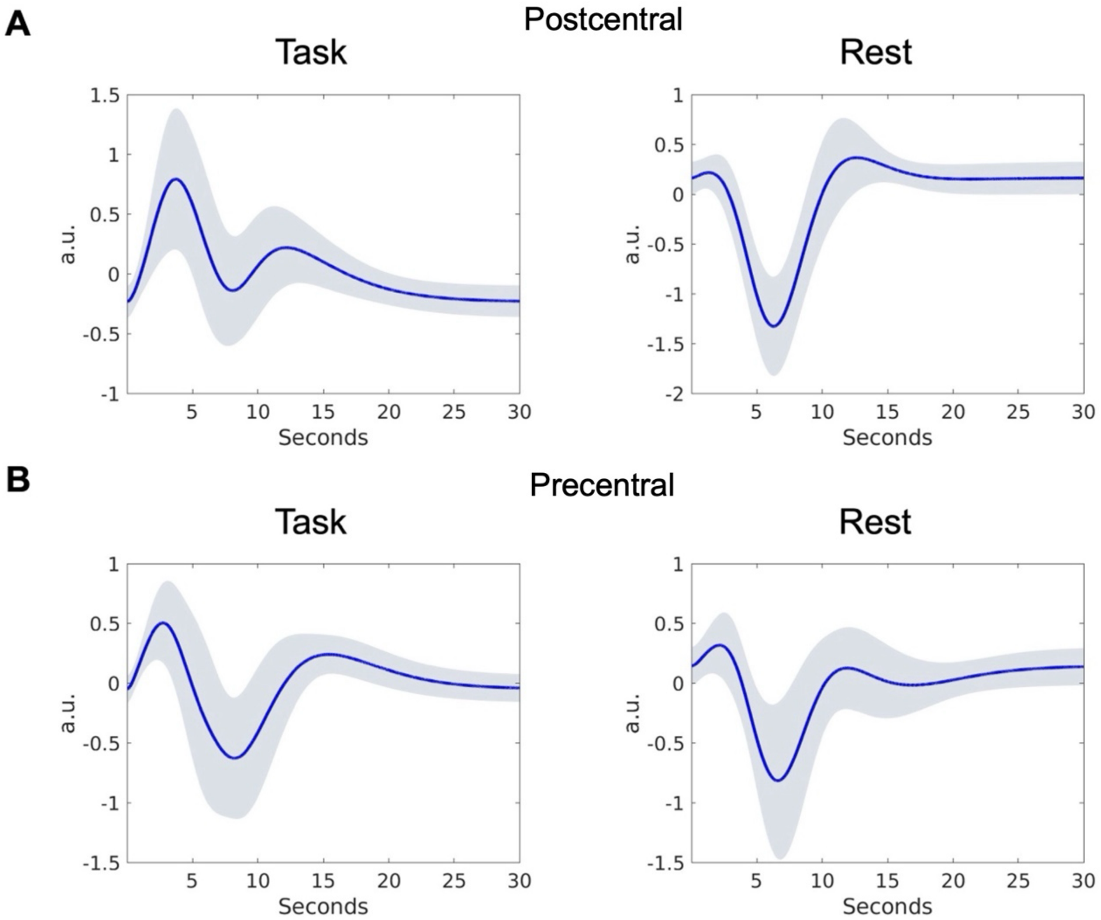

Fig. S8. Average normalized HRF curve using the empirical data during the motor task and rest. The blue curve corresponds to the mean HRF curve across all subjects. The blue shaded area corresponds to the standard error. **(A)** Average normalized HRF curve in the left postcentral region. **(B)** Average normalized HRF curve in the left precentral region. No significant difference was observed on each HRF time point between task and rest.
